# Dirac equation for photons in a fibre: Origin of polarisation

**DOI:** 10.1016/j.heliyon.2024.e28367

**Published:** 2024-03-21

**Authors:** Shinichi Saito

**Affiliations:** Center for Exploratory Research Laboratory, Research & Development Group, Hitachi, Ltd., 1-280 Higashi-Koigakubo, Kokubunji, 185-8601, Tokyo, Japan

**Keywords:** Dirac equation, Klein-Gordon equation, Polarisation, Spin angular momentum, Coherent state, Broken symmetry, Graded index fibre

## Abstract

Spin is a fundamental degree of freedom, which was discovered by Dirac for an electron in his relativistic quantum mechanics, known as the Dirac equation. The origin of spin for a photon is unclear because Maxwell's equations in a vacuum are Lorentz invariant without introducing the concept of spin. Here, the propagation of coherent rays of photons in a graded-index optical fibre is considered to discuss the origin of polarisation for photons using exact solutions of the Laguerre-Gauss and Hermite-Gauss modes. The energy spectrum is massive, and the effective mass is a function of the confinement and orbital angular momentum. The propagation is described by the one-dimensional (1*D*) non-relativistic Schrödinger equation, which is equivalent to the 2*D* space-time Klein-Gordon equation by a unitary transformation. The probabilistic interpretation and the conservation law require the factorisation of the Klein-Gordon equation, leading to the 2*D* Dirac equation with spin. The spin expectation values of photons correspond to the polarisation state on the Poincaré sphere. As an application of the theory, a polarisation interferometer is proposed, whose energy spectrum shows a Dirac cone in the Stokes parameter space.

## 1 Introduction

Dirac elucidated the origin of spin for an electron [1], [2] by unifying quantum mechanics [3], [4], [5] with the theory of relativity [6], which led to the discovery of the Dirac equation [2], [7], [8], [9]. To accept the probabilistic interpretation of the wavefunction together with the conservation law, it was inevitable to factorise the Klein-Gordon equation with a non-trivial spin commutation relationship in a matrix form, called a spinor representation [2], [9]. Therefore, the Lorentz invariance was a major principle behind the existence of spin for an electron. However, the corresponding argument for a photon is not straightforward, since the Maxwell equations are already invariant under the Lorentz transformation [10]. The classical electromagnetic waves, derived from the Maxwell equations, already describe the propagation of light [10], [11], even without introducing quantum mechanics. The polarisation of light is reasonably understood by the transverse nature of electromagnetic radiation [12], [13], [14], [15], but it is less obvious how the quantum mechanical nature of spin for a photon is required to understand the polarisation [16].

Historically, at the early stage of quantum mechanics, there were several attempts [17], [18], [19], [20] to derive a photonic counter part of the Dirac equation. Unfortunately, these works were not quite successful for photons compared with electrons in explaining the origin of polarisation. More recently, spin angular momentum for photons has been revisited in close relationship with optical orbital angular momentum [21], [22], [23], [24], [25], [11], [10], [26], [27], [28], [29] to understand the fundamental quantum mechanical nature of photons [16]. So far, it is widely considered that the total angular momentum is well-defined; however, it is impossible to split it into spin angular momentum and orbital angular momentum [21], [22], [23], [24], [25], [11], [10], [30], [26], [27] in a gauge-invariant way [23], [24], [25], [28], [29]. More recently, there have been several theoretical challenges to accomplish this splitting [31], [32], requiring more time for complete experimental verifications [33], [34], [35], [31], [32]. Therefore, the spin degree of freedom for a photon is not completely well understood in a quantum-mechanical way.

There have been a few previous attempts to derive photonic Dirac equations [36][37][38][39][40][41]. Majorana derived a Dirac-like equation for a photon with an SO(3) commutation relationship [36]. Silveira and Khan also derived Dirac-like equations in 4 × 2 and 8 × 8 matrices, respectively, from Maxwell equations [37][38]. Ohanian discussed the intuitive picture of spin for an electron by comparing the Dirac equation with expressions for spin and orbital angular momentum for a photon [39]. Ohanian claimed that it is no longer necessary to claim an ambiguous internal structure for spin and established the connection between spin and a circularly polarised photon. More recently, Feng and Wu derived a four-vector optical Dirac equation in isotropic inhomogeneous media, which enables the exploration of spin and orbit interaction using the adjoint representation of SO(3) [41]. Despite the progress, a remaining question is why the SU(2) symmetry for spin angular momentum is derived from the U(1) wavefunction of a photon. Even if we accept the general principles of quantum mechanics [1][2][42][43][7][8][9][44], the origin of the SU(2) nature of spin for a photon is not clearly shown, as compared with spin for an electron. In particular, we are interested in how spin for a photon is connected to its macroscopic manifestation as polarisation.

In this paper, we discuss why the spin of a photon is inevitable to understand the propagation in an optical fibre to guarantee the quantum-mechanical probabilistic interpretation, which is based on the same principle for the existence of spin for an electron [1], [2]. We also show how the spin of a photon is linked to the origin of polarisation, as a manifestation of macroscopic coherence for the light from a laser source. To make the argument specific, we restrict our analysis to the propagation of a coherent ray of photons in a GRIN fibre [45], [11], [46], [47], [32], but the extension to the other waveguide will be straightforward. The advantage to employing the GRIN fibre is the availability of the exact solution of the Helmholtz equation, and therefore we can theoretically treat it exactly. We obtain a two-dimensional (2*D*) space-time Dirac equation to describe the propagation of photons along the GRIN fibre, and find that spin is reasonably derived to account for the polarisation state. The rotational invariance of a polarisation state in a waveguide with cylindrical symmetry is a fundamental principle for the quantum nature of spin for photons. For the coherent photons from a laser source, the many-body state is described by a coherent state [26], [48], [49], which is a superposition state made of states with different number of photons. Because of the uncertainty in the number of photons, the phase is coherently fixed, while a macroscopic number of photons occupies the same state, which can be regarded as Bose-Einstein condensation [26], [48], [49], [50], [51], [52], [46], [47], [32]. We introduce the concept of the broken symmetry [53] to photons, which was originally established as the Bardeen-Cooper-Schrieffer (BCS) theory of superconductivity [54], [55], [56], [57], [50], [51], [52]. For photons, the phase coherence is achieved by the onset of lasing, and we show that the energy gap, |Δ|, is opening up due to the broken symmetry as a result of the confinement of photons in a waveguide. It is also well-established that there exists a Boson to recover the symmetry of the system, whose energy should vanish in the long wavelength limit, known as a Nambu-Anderson-Higgs-Goldstone mode [53], [55], [58], [59]. In our case, this corresponds to a radiative mode, escaping from the fibre. By applying the Bogoljubov transformation [60], [56], [57], we show that the phase, *ϕ*, of the order parameter, $\Delta =|\Delta |e^{i\phi }$, corresponds to the azimuthal angle of the polarisation state on the Poincaré sphere.

## 2 Principles

### 2.1 Helmholtz equation

We discuss the propagation of a photon in a material with a refractive index profile of ϵ=ϵ(r) as a function of the position, r=(x,y,z). Note that the wavefunction of a photon and the fundamental equation to describe the wavefunction have not been frequently discussed, as compared with the Schrödinger equation for an electron, except for the pioneering review article of Bialynicki-Birula [40]. A photon is an elementary particle, such that the probability of finding a photon at a certain position (**r**) is described by a wavefunction, Ψ(r), which satisfies the Helmholtz equation [10], [11], [46], [47], [32]

(1) $$\nabla^{2}\Psi (r)=\mu_{0}\epsilon (r)\frac{\partial^{2}}{\partial t^{2}}\Psi (r),$$

where $\nabla =(\partial_{x},\partial_{y},\partial_{z})$, *t* is time, $\mu_{0}$ is the vacuum permeability, and ϵ=ϵ(r) is the profile of the dielectric constant of a material. The values of permeability for most of the materials used in photonics are almost the same as those in a vacuum. In the limit of a vacuum, $\epsilon \to \epsilon_{0}$, where $\epsilon_{0}$ is the vacuum permittivity, the Helmholtz equation becomes

(2) $$\nabla^{2}\Psi (r)=\frac{1}{c^{2}}\frac{\partial^{2}}{\partial t^{2}}\Psi (r),$$

where $c=1/\sqrt{\mu_{0}\epsilon_{0}}$ is the velocity of light in a vacuum. In a vacuum, the dispersion relationship between the angular frequency $\omega_{0}$ and the wavenumber $k_{0}=2\pi /\lambda$ for the wavelength of *λ* becomes linear $\omega_{0}=ck_{0}$, and the wavefunction becomes a simple plane wave, $\Psi (r)=e^{i(k_{0}z-\omega_{0}t)}$, assuming the direction of the propagation is along *z*. The linear dispersion means that a photon does not possess rest mass as an elementary particle [9]. The exploratory research to estimate the limit of the rest mass of a photon has been conducted both experimentally [61][62] and theoretically [63][64]. According to elementary particle physics, we can safely assume that the rest mass of a photon is considered to be negligible or zero in a vacuum for our laser optic experiments. In a vacuum, there is no confinement of a photon, and the mode profile could spread to the entire system. In reality, the mode profile could be adjusted by an optical lens [11] with a diffraction limited mode profile.

On the other hand, in a material with the refractive index profile of $n(r)=\sqrt{\epsilon (r)/\epsilon_{0}}$, a photon tends to propagate in a region where the refractive index is large, to minimise the optical path length, following Fermat's principle and the Eikonal equation [11], [46], [47], [32]. The dispersion relationship ω=ω(k) for the wavenumber, *k*, in a material must be obtained by solving the Helmholtz equation. Depending on n(r), we expect various non-trivial solutions. For example, if n(r) is a periodic function against **r**, the dispersion relationship has a band-gap structure, which is very similar to the electronic band structure for electrons in a material, and the corresponding photonic structure is called a photonic crystal [65], [66], [67], [68].

### 2.2 Quantisation

We assume that the direction of propagation is along *z* and the fibre has cylindrical symmetry. In a GRIN fibre, the refractive index, n(r)=n(r), satisfies

(3) $$n{\left(r\right)}^{2}=n_{0}^{2}\left(1-g^{2}r^{2}\right),$$

where *g* is the parameter to account for the quadratic graded-index profile with the dimension of the inverse of the length, and the radius is defined as $r=\sqrt{x^{2}+y^{2}}$ in a cylindrical coordinate system of (r,ϕ,z). We assume that the solution is decoupled between the direction of propagation and the mode profile, such that the solution is given by a product form,

(4) $$\Psi (r)=\psi (x,y)e^{i(kz-\omega_{0}t)}.$$

By inserting this expression (4) into the Helmholtz equation (1), we obtain

(5) $$\left(\left(\partial_{x}^{2}+\partial_{y}^{2}-\frac{\omega_{0}^{2}g^{2}}{v_{0}^{2}}r^{2}\right)+\left(-k^{2}+\frac{\omega_{0}^{2}}{v_{0}^{2}}\right)\right)\psi (x,y)=0,$$

where $v_{0}=c/n_{0}$ is the velocity of the photon in the uniform media with the refractive index of $n_{0}$. The dispersion relationship is not only a sum along the perpendicular directions, and it must be obtained by solving a quadratic equation, as shown in the following. We will solve this equation with the help of special functions.

In a Cartesian coordinate, the solution of Equation (5) is given [11], [32] by

(6) $$\psi (x,y)=H_{l}\left(\sqrt{2}\frac{x}{w_{0}}\right)H_{m}\left(\sqrt{2}\frac{y}{w_{0}}\right)e^{-\frac{r^{2}}{w_{0}^{2}}},$$

where $w_{0}=\sqrt{2/(gk_{n_{0}})}$ with $k_{n_{0}}=2\pi /\lambda_{n_{0}}=2\pi n_{0}/\lambda =k_{0}n_{0}=\omega_{0}n_{0}/c=\omega_{0}/v_{0}$, where $H_{l}$ and $H_{m}$ are Hermite polynomials and l=0,1,2,⋯ and m=0,1,2,⋯ are quantum numbers to account for the number of nodes along *x* and *y*. This Hermite-Gauss solution of Eq. (6) satisfies

(7) $$\left(\partial_{x}^{2}+\partial_{y}^{2}-\frac{\omega_{0}^{2}g^{2}}{v_{0}^{2}}r^{2}\right)\psi =-2\frac{g}{v_{0}}(l+m+1)\omega_{0}\psi .$$

Considering the cylindrical symmetry of the GRIN fibre, the more reasonable solution of Equation (5) is given by a cylindrical coordinate as,

(8) $$\psi (r,\phi )={\left(\frac{\sqrt{2}r}{w_{0}}\right)}^{\left|m\right|}L_{n}^{\left|m\right|}\left(2{\left(\frac{r}{w_{0}}\right)}^{2}\right)e^{-\frac{r^{2}}{w_{0}^{2}}}e^{im\phi },$$

where $L_{n}^{m}$ is the associated Laguerre polynomial with the radial quantum number of n=0,1,2⋯ and the magnetic angular momentum of m=0,±1,±2,⋯. This Laguerre-Gauss solution satisfies

(9) $$\left(\partial_{r}^{2}+\frac{1}{r}\partial_{r}+\frac{1}{r^{2}}\partial_{\phi }^{2}-\frac{\omega_{0}^{2}g^{2}}{v_{0}^{2}}r^{2}\right)\psi =-2\frac{g}{v_{0}}(2n+|m|+1)\omega_{0}\psi .$$

By assigning l→2n and m→|m|, both Hermite-Gauss and Laguerre-Gauss solutions satisfy the quadratic equation for the dispersion relationship

(10) $$\omega_{0}^{2}-2\delta w_{0}(2n+|m|+1)\omega_{0}-v_{0}^{2}k^{2}=0,$$

where the shift of the angular frequency is defined as $\delta w_{0}=v_{0}g$. By multiplying $\hbar^{2}$, this is also rewritten as

(11) $${\left(\hbar \omega_{0}\right)}^{2}-2(\hbar \delta w_{0})(2n+|m|+1)(\hbar \omega_{0})-{\left(\hbar v_{0}k\right)}^{2}=0.$$

Here, we assumed quantisation conditions for the energy, $E_{0}=\hbar \omega_{0}$, and the momentum, p=ħk. [3], [4], [5], [42], [43], [69], [7], [8], We also defined an energy gap as $\Delta =\hbar \delta w_{0}(2n+|m|+1)=m^{⁎}v_{0}^{2}$, which is similar to the famous Einstein equation $E=mc^{2}$ [70], [71]. In fact, $m^{⁎}$ is the effective mass of the photon in the GRIN fibre, which is not zero in a dispersive GRIN fibre. The effective mass increases upon increasing *n* and |m|, or by the stronger confinement upon increasing *g*. Please note that Δ is finite even at n=0 and m=0, which means that the confinement in the fibre makes a photon massive. By inserting these expressions, the quadratic dispersion equation becomes

(12) $$E_{0}^{2}-2\Delta E_{0}-{\left(v_{0}p\right)}^{2}=0.$$

This equation formally gives two solutions: one for a particle, $E_{0}^{+}=\Delta +\sqrt{\Delta^{2}+{\left(v_{0}p\right)}^{2}}\approx 2\Delta +\frac{{\left(v_{0}p\right)}^{2}}{2\Delta }=2\Delta +\frac{p^{2}}{2m^{⁎}}$, with positive energy. Here, we confirmed that $m^{⁎}$, defined by the energy gap of $\Delta =m^{⁎}v_{0}^{2}$, corresponds to the effective mass due to the quadratic energy dispersion relationship between $E_{0}^{+}$ and *p*, as an asymptotic behaviour in the long wavelength limit. It is reasonable to expect that the solution of the positive energy corresponds to the standard guided optical mode. The other energy branch is given for an anti-particle, $E_{0}^{-}=\Delta -\sqrt{\Delta^{2}+{\left(v_{0}p\right)}^{2}}\approx -\frac{{\left(v_{0}p\right)}^{2}}{2\Delta }=-\frac{p^{2}}{2m^{⁎}}$, with negative energy. The existence of this gapless mode would be linked to the Nambu-Anderson-Higgs-Goldstone mode [53], [55], [58], [59], as shown in the following.

### 2.3 1*D* Schrödinger equation for a photon

In quantum mechanics, energy-time and momentum-position have dualities governed by the uncertainty principle [2], [42], [43], [7], [8]. Then, it is reasonable to expect ${\overset{ˆ}{E}}_{0}=i\hbar \partial_{t}$ and $\overset{ˆ}{p}=(\hbar /i)\partial_{z}$, and consequently, the quadratic dispersion equation becomes

(13) $$i\hbar \partial_{t}e^{ikz-i\omega_{0}t}=-\frac{\hbar^{2}}{2m^{⁎}}\left[\frac{1}{v_{0}^{2}}\partial_{t}^{2}-\partial_{z}^{2}\right]e^{ikz-i\omega_{0}t}.$$

If we define the space-time (2*D*) d'Alembertian as ${□}_{2}=(1/v_{0}^{2})\partial_{t}^{2}-\partial_{z}^{2}$, we obtain

(14) $$i\hbar \partial_{t}\psi_{z}=-\frac{\hbar^{2}}{2m^{⁎}}{□}_{2}\psi_{z},$$

where the wavefunction along *z* is given by a simple plane wave form as $\psi_{z}=e^{ikz-i\omega_{0}t}$. This is essentially the same as the non-relativistic 1*D* Schrödinger equation, although the sign in front of the spatial derivative is opposite. The obtained non-relativistic Schrödinger equation is not symmetric between space and time. In the limit of the vanishing effective mass, $m^{⁎}\to 0$, this becomes the correct form

(15) $${□}_{2}\psi_{z}=0,$$

which gives the massless dispersion of $\omega_{0}=v_{0}k$ with the renormalised velocity of $v_{0}=c/n_{0}$, which is smaller than *c* due to the virtual generations and absorptions of electron-hole pairs during the propagation in a material, as formulated by Feynman diagrams [72]. The application of the special theory of relativity [73], [74] for the moving frame was discussed previously [75].

### 2.4 Nambu-Anderson-Higgs-Goldstone mode

We are considering the coherent ray from a laser source propagating in a GRIN fibre. The propagation is only possible when the phase coherence is achieved due to the pumping above the lasing threshold, otherwise, the phase is randomised and the order parameter, Δ, becomes zero upon averaging over *t*. The situation is very similar to the onset of superconductivity, for which the quasi-particle spectrum becomes massive [54], [53] and a gapless Nambu-Anderson-Higgs-Goldstone mode appears to recover the broken symmetry [53], [55], [58], [59].

In the previous subsection, we obtained the anti-particle gapless solution, such that we discuss the origin of the Nambu-Anderson-Higgs-Goldstone mode [53], [55], [58], [59]. While it is impossible to achieve the propagation in the negative energy unless the time is evolving in a reverse way, it is possible to consider a leaky mode from the fibre by assuming a diverging solution, $e^{-\frac{r^{2}}{w_{0}^{2}}}\to \;e^{+\frac{r^{2}}{w_{0}^{2}}}$, which corresponds to Δ→−Δ. Then, the 1*D* Schrödinger equation becomes

(16) $$-i\hbar \partial_{t}\psi_{z}^{-}=-\frac{\hbar^{2}}{2m^{⁎}}{□}_{2}\psi_{z}^{-},$$

where $\psi_{z}^{-}$ is the wavefunction of the leaky mode. The eigenvalues become $E_{0}^{-\pm }=-\Delta \pm \sqrt{\Delta^{2}+{\left(v_{0}p\right)}^{2}}$, among which one of the solutions provides a positive solution $E_{0}^{-+}=-\Delta +\sqrt{\Delta^{2}+{\left(v_{0}p\right)}^{2}}\approx \frac{{\left(v_{0}p\right)}^{2}}{2\Delta }=\frac{p^{2}}{2m^{⁎}}$. This dispersion is gapless, which means an arbitrary small energy is required to recover the symmetry. For propagation in a fibre, the symmetry is broken because of the optical confinement in the fibre. The gapless mode guarantees the existence of a mode to recover the confinement by escaping from the fibre. In our case, the so-called Nambu-Goldstone Boson is also made of a photon as a branch for leaking from the fibre. In reality, the leaky mode is completely different in energy and momentum, such that the guided mode is not affected by the leaky mode at all. Nevertheless, it is theoretically interesting to consider the fundamental principle behind the photon propagation in the GRIN fibre.

If we use the same notation for the leaky mode, the wavefunction of the guided mode, $\psi_{z}^{+}$, must satisfy the time-reversal 1*D* Schrödinger equation

(17) $$i\hbar \partial_{t}\psi_{z}^{+}=-\frac{\hbar^{2}}{2m^{⁎}}{□}_{2}\psi_{z}^{+},$$

which will give two formal solutions $E_{0}^{+\pm }=\Delta \pm \sqrt{\Delta^{2}+{\left(v_{0}p\right)}^{2}}$ for the standard time evolution and time-reversal evolution, respectively.

### 2.5 2*D* Klein-Gordon equation

Equation (17) is the same form as the standard non-relativistic Schrödinger equation [7], [8]. This will give an eigenvalue of $E_{0}^{+\pm }=\Delta \pm \sqrt{\Delta^{2}+{\left(v_{0}p\right)}^{2}}$. We also obtained the complex conjugate equation for the mode propagating in the opposite direction,

(18) $$-i\hbar \partial_{t}\psi_{z}^{-}=-\frac{\hbar^{2}}{2m^{⁎}}{□}_{2}\psi_{z}^{-},$$

whose eigenvalue is $E_{0}^{-\pm }=-\Delta \pm \sqrt{\Delta^{2}+{\left(v_{0}p\right)}^{2}}$. The existence of the conjugate solution is guaranteed by the time-reversal symmetry (t↔−t) of the system $\psi_{z}^{+}={\left(\psi_{z}^{-}\right)}^{†}$ and $\psi_{z}^{-}={\left(\psi_{z}^{+}\right)}^{†}$.

Here, we are considering a coherent ray of photons emitted from a laser source. The mode is confined in a GRIN fibre, such that the original rotational and translational symmetries in free space are broken. The shift of the energy of ±Δ could be interpreted as an energy shift of the vacuum upon lasing because the laser operation is similar to the Bose-Einstein condensation with a macroscopic number of photons occupying the same state. According to the theory of superconductivity [54], [56], the energy shift for superconducting condensation is $N_{e}D(E_{F})|\Delta {|}^{2}/2$, where $N_{e}$ is the number of electrons, $D(E_{F})$ is the density of electrons per energy, and |Δ| is the energy gap. For electrons, due to Pauli's exclusion principle, the number of electrons affected by the superconducting pairing interaction is limited to those electrons near the Fermi energy, $E_{F}$. On the other hand, photons are Bose particles, so they can occupy the same state. Consequently, we expect the total energy change of N|Δ| for photons in a laser, where N is the number of photons in a cavity. To compensate for this energy shift by condensation, we consider a unitary transformation of the wavefunction, $\psi_{z}=e^{\frac{i}{\hbar }\Delta t}\psi_{z}^{+}$, which yields

(19) $$\partial_{t}\psi_{z}^{+}=-\frac{i}{\hbar }\Delta e^{-\frac{i}{\hbar }\Delta t}\psi_{z}+e^{-\frac{i}{\hbar }\Delta t}\partial_{t}\psi_{z}$$

(20) $$\partial_{t}^{2}\psi_{z}^{+}=-\frac{\Delta^{2}}{\hbar^{2}}\Delta e^{-\frac{i}{\hbar }\Delta t}\psi_{z}-2\frac{i}{\hbar }\Delta e^{-\frac{i}{\hbar }\Delta t}\partial_{t}\psi_{z}+e^{-\frac{i}{\hbar }\Delta t}\partial_{t}^{2}\psi_{z}.$$

By inserting Equations (19) and (20) into the Schrödinger equation (17), we obtain the 2*D* Klein-Goldon equation [9],

(21) $$\left[\frac{1}{v_{0}^{2}}\partial_{t}^{2}-\partial_{z}^{2}+\frac{m^{⁎2}v_{0}^{2}}{\hbar^{2}}\right]\psi_{z}=0,$$

for which the solution is $\psi_{z}=e^{ikz-i\omega t}$ with an eigenvalue of $E=\hbar \omega =\pm \sqrt{\Delta^{2}+{\left(v_{0}p\right)}^{2}}$. The U(1) unitary transformation is only a constant shift of the energy, which is considered to be attributed to the difference of vacuum energies due to the difference of symmetries between a 1*D* confined GRIN fibre and free space. The energy gap of Δ, to be responsible for the finite effective mass of $m^{⁎}$, cannot be removed by this unitary transformation to account for the gauge invariance.

Similarly, we also consider the unitary transformation, $\psi_{z}=e^{-\frac{i}{\hbar }\Delta t}\psi_{z}^{-}$, which yields

(22) $$\partial_{t}\psi_{z}^{-}=+\frac{i}{\hbar }\Delta e^{+\frac{i}{\hbar }\Delta t}\psi_{z}+e^{+\frac{i}{\hbar }\Delta t}\partial_{t}\psi_{z}$$

(23) $$\partial_{t}^{2}\psi_{z}^{-}=-\frac{\Delta^{2}}{\hbar^{2}}\Delta e^{+\frac{i}{\hbar }\Delta t}\psi_{z}+2\frac{i}{\hbar }\Delta e^{+\frac{i}{\hbar }\Delta t}\partial_{t}\psi_{z}+e^{+\frac{i}{\hbar }\Delta t}\partial_{t}^{2}\psi_{z}.$$

By inserting Equations (22) and (23) into the Schrödinger equation (18), we obtain the same results as the Klein-Gordon equation (21) for the same wavefunction $\psi_{z}=e^{ikz-i\omega t}$ and the energy spectrum $E=\hbar \omega =\pm \sqrt{\Delta^{2}+{\left(v_{0}p\right)}^{2}}$.

Alternatively, the 2*D* Klein-Gordon equation could be obtained from the spectrum of $E=\pm \sqrt{\Delta^{2}+{\left(v_{0}p\right)}^{2}}$, while assuming the quantisation conditions $\overset{ˆ}{E}=\hbar \omega =i\hbar \partial_{t}$, $\overset{ˆ}{p}=\hbar k=-i\hbar \partial_{z}$, and $\Delta =m^{⁎}v_{0}^{2}$. Inserting these into $E^{2}=\Delta^{2}+{\left(v_{0}p\right)}^{2}$, we obtain

(24) $$-\hbar^{2}\partial_{t}^{2}\psi_{z}=\left[m^{⁎2}v_{0}^{4}-v_{0}^{2}\hbar^{2}\partial_{z}^{2}\right]\psi_{z},$$

which is in agreement with the Klein-Gordon equation (21). Therefore, the massive feature of the propagation for a photon in a GRIN fibre along the direction of propagation is essentially described by the Klein-Gordon equation.

### 2.6 2*D* Dirac equation

It is well-established for an electron that the Klein-Gordon equation is not appropriate to consider the quantum-mechanical probabilistic interpretation with the conservation law [2], [9]. To cope with this problem, Dirac introduced spinor matrix operators, which enabled the factorisation of the Klein-Gordon equation only like a primitive mathematical formula of $x^{2}-y^{2}=(x+y)(x-y)$, leading to the Dirac equation and the derivation of spin as an inherent quantum degree of freedom [2], [9]. We apply the same technique to our 2*D* Klein-Gordon equation (21) for a photon in a GRIN fibre. We assume

(25) $$\left(\frac{1}{v_{0}}\partial_{t}-\alpha_{z}\partial_{z}-\alpha_{x}i\frac{m^{⁎}v_{0}}{\hbar }\right)\left(\frac{1}{v_{0}}\partial_{t}+\alpha_{z}\partial_{z}+\alpha_{x}i\frac{m^{⁎}v_{0}}{\hbar }\right)\psi_{z}=0,$$

where $\alpha_{x}$ and $\alpha_{z}$ are parameters to satisfy the Klein-Gordon equation (21). After the expansion, we obtain

(26) $$\left[\frac{1}{v_{0}^{2}}\partial_{t}^{2}-\alpha_{z}^{2}\partial_{z}^{2}+\frac{m^{⁎2}v_{0}^{2}}{\hbar^{2}}\alpha_{x}^{2}-i\frac{m^{⁎}v_{0}}{\hbar }(\alpha_{z}\alpha_{x}+\alpha_{x}\alpha_{z})\right]\psi_{z}=0.$$

By comparing this with the Klein-Gordon equation, we obtain $\alpha_{x}^{2}=1$, $\alpha_{z}^{2}=1$, and $\alpha_{z}\alpha_{x}+\alpha_{x}\alpha_{z}=0$. To satisfy these equations, in particular for the last anti-commutation relationship, we obtain

(27) $$\alpha_{x}=\sigma_{x}=\left(\begin{array}{cc} 0\; & 1\\ 1\; & 0 \end{array}\right)$$

(28) $$\alpha_{z}=\sigma_{z}=\left(\begin{array}{cc} 1\; & 0\\ 0\; & -1 \end{array}\right),$$

where $\sigma_{i}$ (i=x,y,z) is the Pauli spin matrices [2], [7], [8]. As shown later, the other choices of spin operators are possible, due to the rotational symmetries of spin states.

Finally, we obtain the 2*D* Dirac equation for a photon by multiplying *ħ* as

(29) $$\left(i\hbar \frac{1}{v_{0}}\partial_{t}-i\hbar \sigma_{z}\partial_{z}+\sigma_{x}m^{⁎}v_{0}\right)\left(i\hbar \frac{1}{v_{0}}\partial_{t}+i\hbar \sigma_{z}\partial_{z}-\sigma_{x}m^{⁎}v_{0}\right)\psi_{z}=0.$$

This indicates decoupled forms of

(30) $$\left(i\hbar \frac{1}{v_{0}}\partial_{t}-i\hbar \sigma_{z}\partial_{z}+\sigma_{x}m^{⁎}v_{0}\right)\psi_{z}=0,$$

and

(31) $$\left(i\hbar \frac{1}{v_{0}}\partial_{t}+i\hbar \sigma_{z}\partial_{z}-\sigma_{x}m^{⁎}v_{0}\right)\psi_{z}=0.$$

The wavefunction of the 2*D* Dirac equation is described by the 2-component spinor representation as

(32) $$\psi_{z}=e^{ikz-i\omega t}\left(\begin{array}{c} \chi_{↑}\\ \chi_{↓} \end{array}\right),$$

where the spin up and down components correspond to the left- and right-circular polarisation states [46], respectively, and the spinor components are $\chi_{↑}=\langle \;↑|\psi_{z}\rangle$ and $\chi_{↓}=\langle \;↓|\psi_{z}\rangle$. Consequently, by assuming the proper factorisation of the corresponding 2*D* Dirac equation, we have derived the spin of a photon from the Helmholtz equation, which is assigned for the polarisation degree of freedom.

By inserting the plane wave solution along the direction of the propagation (*z*), we obtain

(33) $$\left(i\hbar \partial_{t}+v_{0}p\sigma_{z}+\sigma_{x}m^{⁎}v_{0}^{2}\right)\psi_{z}=0$$

(34) $$\left(i\hbar \partial_{t}-v_{0}p\sigma_{z}-\sigma_{x}m^{⁎}v_{0}^{2}\right)\psi_{z}=0$$

which are equations of motion to describe the time evolution of spin. Here, we put $\xi =v_{0}p$ and $\Delta =m^{⁎}v_{0}^{2}$, and they become

(35) $$i\hbar \partial_{t}\psi_{z}=-h\cdot \sigma \psi_{z}$$

(36) $$i\hbar \partial_{t}\psi_{z}=h\cdot \sigma \psi_{z},$$

where the effective *“magnetic”* field is h=(Δ,0,ξ) in the unit of energy, and the spin/polarisation vector is $\sigma =(\sigma_{x},\sigma_{y},\sigma_{z})$. Note that the two conjugate equations are obtained in pairs, which correspond to the opposite magnetic fields to each other. This is guaranteed by the time-reversal symmetry of the system, such that we should have two solutions, which are conjugate to each other.

In the matrix form, the equations of motion are rewritten as

(37) $$\hbar \omega \left(\begin{array}{c} \chi_{↑}\\ \chi_{↓} \end{array}\right)=-\left(\begin{array}{cc} \xi \; & \Delta \\ \Delta \; & -\xi \end{array}\right)\left(\begin{array}{c} \chi_{↑}\\ \chi_{↓} \end{array}\right)$$

(38) $$\hbar \omega \left(\begin{array}{c} \chi_{↑}\\ \chi_{↓} \end{array}\right)=+\left(\begin{array}{cc} \xi \; & \Delta \\ \Delta \; & -\xi \end{array}\right)\left(\begin{array}{c} \chi_{↑}\\ \chi_{↓} \end{array}\right),$$

whose eigenvalues are $E_{\pm }=\pm \sqrt{\xi^{2}+\Delta^{2}}$.

We define the Hamiltonian for the left and right polarising states as

(39) $$H_{L}=h\cdot \sigma =\left(\begin{array}{cc} \xi \; & \Delta \\ \Delta \; & -\xi \end{array}\right)$$

(40) $$H_{R}=-h\cdot \sigma =-\left(\begin{array}{cc} \xi \; & \Delta \\ \Delta \; & -\xi \end{array}\right),$$

then, the 2*D* Dirac equation can be rewritten as

(41) $$\left[i\hbar \partial_{t}-H_{L}\right]\left[i\hbar \partial_{t}-H_{R}\right]\psi_{z}=0.$$

Equivalently, we can also re-write

(42) $$\left[i\hbar \partial_{t}-H\right]\left[-i\hbar \partial_{t}-H\right]\psi_{z}=0,$$

where $H=H_{L}$, emphasising the time-reversal symmetry and the Hermitian nature of the Hamiltonian $H^{†}=H$.

### 2.7 Polarisation state

The detailed discussions for a photon are provided in the supplementary information, and here, we will summarise our results on the 2*D* Dirac equation. We started from the Helmholtz equation in a GRIN fibre and modified it to the 1*D* Schrödinger equation and the 2*D* Klein-Gordon equation. Then, we realised a photon in a GRIN fibre is massive due to the confinement and the orbital angular momentum, which is described by the complex energy gap, $\Delta e^{i\phi }$. It is well-known that the massive quantum particle, described by the Klein-Gordon equation, cannot satisfy relativistic covariance due to the second-order derivatives [9]. We have imposed the factorisation requirement in exactly the same way to derive the Dirac equation for an electron [9]. This requirement comes from the relativistic covariance for time (*t*) and space (*z*), and the relativistic equation must be first order [9]. Then, the SU(2) commutation relationships of Pauli spin matrices were reasonably introduced to obtain the decoupled 2*D* Dirac equations for a photon

(43) $$\left[i\hbar \partial_{t}-H_{L}\right]\psi_{z}^{L}=0$$

(44) $$\left[i\hbar \partial_{t}-H_{R}\right]\psi_{z}^{R}=0,$$

where $\psi_{z}^{L}$ and $\psi_{z}^{R}$ are wavefunctions for left and right circularly polarised states, and we confirmed the time-reversal symmetry of $H_{R}=-H_{L}$. In a steady state, the Hamiltonians must be diagonalised to have energies $E=\hbar \omega =\pm \sqrt{\xi^{2}+\Delta^{2}}$ for forward (t>0) and backward (t<0) propagations over time, respectively. For standard forward propagation, we take the positive sign, $E=\hbar \omega =+\sqrt{\xi^{2}+\Delta^{2}}$, for k>0. After diagonalisation, we have renormalised the chiral states, |L〉=(1,0) and |R〉=(0,1), whose quantisation axis is locked along the direction of the propagation, and a general spin state with the polar angle of *θ* and the azimuthal angle *ϕ* is given by the superposition state

(45) $$|\theta ,\phi \rangle =\left(\begin{array}{c} e^{-i\frac{\phi }{2}}\cos\left(\frac{\theta }{2}\right)\\ e^{+i\frac{\phi }{2}}\sin\left(\frac{\theta }{2}\right) \end{array}\right)=e^{-i\frac{\phi }{2}}\cos\left(\frac{\theta }{2}\right)|L\rangle +e^{+i\frac{\phi }{2}}\sin\left(\frac{\theta }{2}\right)|R\rangle .$$

We must be careful when considering superposition states only among states with the same energy. By enabling the superposition state, the spin of a photon can point in any direction, and the rotational symmetry of the polarisation is recovered.

### 2.8 Coherent macroscopic wavefunction

Finally, we briefly describe the coherent many-body state of photons in a ray propagating along a GRIN fibre emitted from a laser source [11], [46], [32]. Here, we employ the chiral representation using the Left-Right (LR)-basis, but we can also use other representations like the Horizontal-Vertical (HV) basis [11], [46], [32]. The direction of the polarisation axis is not fixed solely from the Dirac equation. However, in comparison with the electric field obtained by using the corresponding coherent state, we can assign that the chiral axis of $S_{3}$ in the polarisation state is locked into the direction of the propagation [46]. We use our convention that the left-circular polarised state is located at $S_{3}=1$ in the Stokes parameter notation, normalised to be unity in magnitude [46]. In this convention, the phase front of the electric field at the left-circular state is rotating towards the left, which is anti-clock wise, seen from the detector side, using the standard right-handed (x,y,z) coordinate system [46]. Of course, any other convention is acceptable as long as the whole argument is closed consistently.

In the previous section, we confirmed that the dispersion relationship does not depend on the polarisation state in a GRIN fibre. Therefore, the energy of photons is independent of the polarisation state, and photons can take any polarisation state, which is not determined solely from the fundamental Helmholtz equation or the Dirac equation. This is a generic feature for all physical variables, as a solution of a differential equation requires boundary conditions such as an initial condition for the time evolution. For the photon at the eigenstate, propagating in the GRIN fibre, there is no time-evolution for the polarisation state, such that the initial polarisation state will be maintained. We discuss how the polarisation state is described in a many-body state of photons.

We consider the existence of a single mode in a multi-mode fibre, but for the application of the multi-modes, we only need to consider the superposition of available modes, so the extension is quite straightforward. Photons are Bosons, so multiple photons can occupy the same state. If a macroscopic number of Bosons is occupying the same state, the macroscopic Bosons are described by a single wavefunction, and the corresponding macroscopic coherent assembly is called Bose-Einstein condensate [72], [57], [52], [50]. Photons emitted from a laser source are also considered to be residing in such a Bose-Einstein condensed state, in which phase coherence is expected because a macroscopic number of photons are occupying the same state, described by one macroscopic wavefunction. As shown in the following, the wavefunction is nothing but the spinor wavefunction describing the polarisation state of photons. To maintain phase coherence, we must enable the fluctuation of the number of photons, since the number and the phase are conjugate to each other. This is achieved by considering the coherent states of photons [26], [48], [49],

(46) $$|\alpha_{L}\rangle =e^{-\frac{|\alpha_{L}{|}^{2}}{2}}e^{\alpha_{L}{\overset{ˆ}{a}}_{L}^{†}}|0\rangle$$

(47) $$|\alpha_{R}\rangle =e^{-\frac{|\alpha_{R}{|}^{2}}{2}}e^{\alpha_{R}{\overset{ˆ}{a}}_{R}^{†}}|0\rangle ,$$

for left- and right-circular polarised states, respectively. Here, we have defined the creation operator of ${\overset{ˆ}{a}}_{\sigma }^{†}$ and annihilation operator of ${\overset{ˆ}{a}}_{\sigma }$ for the left and right polarisation states, σ=L and σ=R, which satisfy the Bose commutation relationships $[{\overset{ˆ}{a}}_{\sigma },{\overset{ˆ}{a}}_{\sigma^{\prime }}]=0$ and $[{\overset{ˆ}{a}}_{\sigma },{\overset{ˆ}{a}}_{\sigma^{\prime }}^{†}]=\delta_{\sigma ,\sigma^{\prime }}$, using the Kronecker delta of $\delta_{\sigma ,\sigma^{\prime }}$ [9], [26], [48], [49], [46], [32]. The coherent states [9], [26], [48], [49], [46], [32] are characterised by the complex number of $\alpha_{\sigma }$, which is an eigenvalue of the annihilation operator, as

(48) $${\overset{ˆ}{a}}_{\sigma }|\alpha_{\sigma }\rangle =\alpha_{\sigma }|\alpha_{\sigma }\rangle ,$$

whose conjugate becomes

(49) $$\langle \alpha_{\sigma }|{\overset{ˆ}{a}}_{\sigma }^{†}=\langle \alpha_{\sigma }|\alpha_{\sigma }^{⁎},$$

where $\alpha_{\sigma }^{⁎}$ is the complex conjugate of $\alpha_{\sigma }$. Consequently, it is straightforward to obtain the quantum-mechanical average of the number operator

(50) $$\langle \alpha_{\sigma }|{\overset{ˆ}{a}}_{\sigma }^{†}{\overset{ˆ}{a}}_{\sigma }|\alpha_{\sigma }\rangle =|\alpha_{\sigma }{|}^{2},$$

which is the number of photons, $N_{\sigma }$, in the polarisation state of *σ*, and we have a sum rule for the total number of $N=N_{L}+N_{R}$.

Now, we can assign the phases of the wavefunction to the polarisation state. The polarisation state of a photon is characterised by the polar angle of *θ* and the azimuthal angle of *ϕ* in the Poincare sphere (Suppl. Fig. 5) [76], [77], [78], [11], [7], [8], [46]. In the macroscopic coherent state, this could be implemented by assigning

(51) $$\alpha_{L}=\sqrt{N}e^{-i\frac{\phi }{2}}\cos\left(\frac{\theta }{2}\right)$$

(52) $$\alpha_{R}=\sqrt{N}e^{+i\frac{\phi }{2}}\sin\left(\frac{\theta }{2}\right).$$

Consequently, we obtain the many-body wavefunction as

(53) $$|\alpha_{L},\alpha_{R}\rangle =|\alpha_{L}\rangle |\alpha_{R}\rangle =e^{-\frac{N}{2}}e^{\alpha_{L}{\overset{ˆ}{a}}_{L}^{†}}e^{\alpha_{R}{\overset{ˆ}{a}}_{R}^{†}}|0\rangle$$

(54) $$=e^{-\frac{N}{2}}\exp\left[\sqrt{N}e^{-i\frac{\phi }{2}}\cos\left(\frac{\theta }{2}\right){\overset{ˆ}{a}}_{L}^{†}\right]\exp\left[\sqrt{N}e^{+i\frac{\phi }{2}}\sin\left(\frac{\theta }{2}\right){\overset{ˆ}{a}}_{R}^{†}\right]|0\rangle .$$

Note that the entire wavefunction is characterised by single-particle wavefunction with parameters *θ* and *ϕ*.

We can confirm the spinor representation of the single-particle wavefunction for the polarisation state in this many-body wavefunction simply by applying the complex electric field operator,

(55) $$\overset{ˆ}{E}(r,t)=\sqrt{\frac{2\hbar \omega_{0}}{\epsilon V}}e^{ikz-i\omega_{0}t}\left({\overset{ˆ}{a}}_{L}\overset{ˆ}{l}+{\overset{ˆ}{a}}_{R}\overset{ˆ}{r}\right),$$

where $V=w_{0}^{2}L_{z}$ with the length of $L_{z}$ for the fibre along *z*, $\overset{ˆ}{l}=(\overset{ˆ}{x}+i\overset{ˆ}{y})/\sqrt{2}$ and $\overset{ˆ}{r}=(\overset{ˆ}{x}-i\overset{ˆ}{y})/\sqrt{2}$ are complex unit vectors for directions of left and right polarisation states for the unit vectors of the coordinate of $\overset{ˆ}{x}$ and $\overset{ˆ}{y}$ along *x* and *y*. We obtain

(56) $$\overset{ˆ}{E}(r,t)|\alpha_{L},\alpha_{R}\rangle =\sqrt{\frac{2\hbar \omega }{\epsilon V}}\Psi (r,t)\left(\begin{array}{c} e^{-i\frac{\phi }{2}}\cos\left(\frac{\theta }{2}\right)\\ e^{+i\frac{\phi }{2}}\sin\left(\frac{\theta }{2}\right) \end{array}\right)|\alpha_{L},\alpha_{R}\rangle ,$$

to confirm $|\alpha_{L},\alpha_{R}\rangle$ is the eigenstate for $\overset{ˆ}{E}(r,t)$, and the polarisation state is described by the Bloch state

(57) $$|\mathrm{Bloch}\rangle =\left(\begin{array}{c} e^{-i\frac{\phi }{2}}\cos\left(\frac{\theta }{2}\right)\\ e^{+i\frac{\phi }{2}}\sin\left(\frac{\theta }{2}\right) \end{array}\right),$$

which is the solution (45) |θ,ϕ〉 of the Dirac equation for a single photon.

### 2.9 Stokes parameters

We obtained the macroscopic quantum-mechanical wavefunction using the coherent state for photons. We can calculate relevant observables such as electric field, magnetic field, and so on as expectation values of this wavefunction [46], [32]. Here, we show that the spin expectation values are the Stokes parameters [46], [32] on the Poincaré sphere by using |θ,ϕ〉.

The many-body spin operators [46], [32] for photons in the LR basis are defined as ${\overset{ˆ}{S}}_{x}=\hbar {\overset{ˆ}{\psi }}_{\mathrm{LR}}^{†}\sigma_{1}{\overset{ˆ}{\psi }}_{\mathrm{LR}}$, ${\overset{ˆ}{S}}_{y}=\hbar {\overset{ˆ}{\psi }}_{\mathrm{LR}}^{†}\sigma_{2}{\overset{ˆ}{\psi }}_{\mathrm{LR}}$, and ${\overset{ˆ}{S}}_{z}=\hbar {\overset{ˆ}{\psi }}_{\mathrm{LR}}^{†}\sigma_{3}{\overset{ˆ}{\psi }}_{\mathrm{LR}}$, the spinor representation of the creation and annihilation field operators are defined as

(58) $${\overset{ˆ}{\psi }}_{\mathrm{LR}}^{†}=({\overset{ˆ}{a}}_{L}^{†},{\overset{ˆ}{a}}_{R}^{†})$$

(59) $${\overset{ˆ}{\psi }}_{\mathrm{LR}}=\left(\begin{array}{c} {\overset{ˆ}{a}}_{L}\\ {\overset{ˆ}{a}}_{R} \end{array}\right).$$

The spin operators are also rewritten as ${\overset{ˆ}{S}}_{x}=\hbar \left({\overset{ˆ}{a}}_{L}^{†}{\overset{ˆ}{a}}_{R}+{\overset{ˆ}{a}}_{R}^{†}{\overset{ˆ}{a}}_{L}\right)$, ${\overset{ˆ}{S}}_{y}=\hbar \left(-i{\overset{ˆ}{a}}_{L}^{†}{\overset{ˆ}{a}}_{R}+i{\overset{ˆ}{a}}_{R}^{†}{\overset{ˆ}{a}}_{L}\right)$, and ${\overset{ˆ}{S}}_{z}=\hbar \left({\overset{ˆ}{n}}_{L}-{\overset{ˆ}{n}}_{R}\right)$. Therefore, it is straightforward to calculate the quantum-mechanical expectation value as $\langle {\overset{ˆ}{S}}_{x}\rangle =\hbar \left(\alpha_{L}^{⁎}\alpha_{R}+\alpha_{R}^{⁎}\alpha_{L}\right)=\hbar N\cos\phi \sin\theta$, $\langle {\overset{ˆ}{S}}_{y}\rangle =\hbar \left(-i\alpha_{L}^{⁎}\alpha_{R}+i\alpha_{R}^{⁎}\alpha_{L}\right)=\hbar N\sin\phi \sin\theta$, $\langle {\overset{ˆ}{S}}_{z}\rangle =\hbar \left(N_{L}-N_{R}\right)=\hbar N\cos\theta$. For the total number of photons, $S_{0}$ is defined as ${\overset{ˆ}{S}}_{0}=\hbar \psi_{\mathrm{LR}}^{†}1\psi_{\mathrm{LR}}=\hbar ({\overset{ˆ}{a}}_{L}^{†}{\overset{ˆ}{a}}_{L}+{\overset{ˆ}{a}}_{R}^{†}{\overset{ˆ}{a}}_{R})$, and its expectation value for the coherent state becomes $\langle {\overset{ˆ}{S}}_{0}\rangle =\hbar N$.

We define the total spin vector operator as $\overset{ˆ}{S}=({\overset{ˆ}{S}}_{0},{\overset{ˆ}{S}}_{x},{\overset{ˆ}{S}}_{y},{\overset{ˆ}{S}}_{z})$, and its expectation value by the coherent state is given by the Stokes parameters [76], [77], [78], [11], [12], [13], [14], [15], [79], [80], [81], [82], [83], [84], [85], [46], [32]

(60) $$S=\langle \overset{ˆ}{S}\rangle =\left(\begin{array}{c} S_{0}\\ S_{1}\\ S_{2}\\ S_{3} \end{array}\right)=\hbar N\left(\begin{array}{c} 1\\ \sin\theta \cos\phi \\ \sin\theta \sin\phi \\ \cos\theta \end{array}\right).$$

More generally, a ray in the fibre could contain a contribution from the incoherent state [76], [77], [11], [12], [13]. The phase degrees of freedom described by phases of *θ* and *ϕ* are responsible for the coherent parts, which are included in $S_{1}$, $S_{2}$, and $S_{3}$, while the total number of photons of N is responsible for both contributions, included in $S_{0}$. Therefore, we can define the degree of polarisation to measure the coherence as a measure of polarisation,

(61) $$d_{p}=\frac{\sqrt{S_{1}^{2}+S_{2}^{2}+S_{3}^{2}}}{S_{0}},$$

which takes the value from 0 (unpolarised) to 1 (fully polarised). Similarly, we can also define the degree of unpolarised light as

(62) $$d_{u}=\frac{S_{0}-\sqrt{S_{1}^{2}+S_{2}^{2}+S_{3}^{2}}}{S_{0}}=1-d_{p},$$

which takes the value from 0 (fully polarised) to 1 (unpolarised). The amount of polarised light ($d_{p}$) plays a role of an order parameter to measure the quantum coherence [11], [12], [13], [86].

## 3 Discussions

### 3.1 Comparison with previous works

Most of the previous works to derive Dirac-like equations for photons were based on the assumption that a photon is propagating in a vacuum [17][18][19][20][36][37][38][39][40][41][87][88][71], while we have considered a GRIN fibre, where exact solutions are available. There is a remarkable difference from previous works that a photon in the GRIN fibre is massive, characterised by an energy gap in the dispersion relationship. Consequently, we could apply the same relativistic principle for a photon in the fibre as for an electron to derive the Dirac equation [9]. The spin of a photon in the fibre was reasonably described by the SU(2) wavefunction, and we can describe the arbitrary spin state by the superposition state of left and right circularly polarised states. Another difference is whether the derivation is based on the SO(3) symmetry [36][41] or the SU(2) symmetry. There is no difference between SO(3) and SU(2) for calculating the expectation values of spin components; however, there is a difference if we consider the geometrical phases [89][90][91][92][93]. We believe it is reasonable to use the SU(2) symmetry for describing the complex wavefunction of a 2-level system, and our formulation is suitable to explore the impact of a geometrical phase. In fact, we have recently demonstrated that the phase between orthogonal polarised states could be controlled by the proposed Poincar'e rotator, which relies on the phase difference of SU(2) upon propagation [94][95][96].

In the pioneering work of Ohanian [39], the intuitive correlation between the classical description of the angular momentum of a photon and the Dirac equation was discussed. Ohanian claimed that it is possible to give up the ambiguous internal degree of freedom for the spin of an electron, since spin is correlated with the picture of circularly polarised waves. However, we have recently shown that the only chiral component of angular momentum could be obtained by the classical correspondence of angular momentum [32]. In the present context, we have obtained only left and right circularly polarised states directly from the decoupled Dirac equations. The arbitrary spin state could be achieved by enabling the superposition state between left and right circularly polarised states. Therefore, we still need to accept the general principles of quantum mechanics, and we think that the spin of a photon originates from an internal degree of freedom of a photon as an elementary particle. It is beyond the scope of this work to establish a more intuitive feature of spin outside the current framework of quantum mechanics. Our approach is to accept modern quantum mechanics and relativity for understanding the spin of a photon. Nevertheless, we believe our approach is a reasonable extension of the derivation of the Dirac equation for a photon, and we establish that it is inevitable for a photon to have spin as an internal degree of freedom with the SU(2) symmetry to follow the covariance in the first order. Our conclusion is based on the standard assumption that modern quantum mechanics is more fundamental than classical mechanics. The present work shows that we can understand the spin of a photon on the basis of standard quantum mechanics at least in a fibre due to the confinement. We also believe the revelation that the energy gap to describe the confinement has another internal degree of freedom of orbital angular momentum, as discussed in the following. Upon understanding that the spin of a photon originates from the quantum-mechanical internal degree of freedom, it is also possible to understand polarisation as coherent states of photons with a broken rotational symmetry, as discussed in the following.

### 3.2 Broken symmetry

As previously stated, the coherent state of photons emitted from a laser source is considered to be characterised by the broken SU(2) state with fixed phases of *θ* and *ϕ*. In the original Helmholtz equation and the Dirac equation for photons, we have confirmed the rotational symmetry expected for the GRIN fibre considered in this paper. Nevertheless, in the coherent state, the superposition state with a different number of photons is allowed, and the phases are fixed as a result of Bose-Einstein condensation. We have developed a fundamental theory to understand the quantum-mechanical origin of spin for photons in close analogy with the theory of superconductivity [54], [55], [56], [57], [53], [60], [97]. Here, we discuss the similarity and the difference in more detail.

The most important difference arises from the statistics of the elementary particles involved. Note that superconductivity is driven by electrons, which are Fermions [54], [56], and therefore, it is hard to expect similarity to Bose-Einstein condensation. Nevertheless, the weak attractive interaction mediated by phonons or spin fluctuations could lead to the formation of a Cooper pair, which is made by an electron with the wavevector **k** and spin up (↑), and its time-reversal symmetric counterpart of −k and spin down (↓) electron. The entire system is described by the BCS variational wavefunction

(63) $$|\phi \rangle =\prod_{k}\left[u_{k}+v_{k}{\overset{ˆ}{c}}_{k↑}^{†}{\overset{ˆ}{c}}_{-k↓}^{†}\right]|0\rangle ,$$

where ${\overset{ˆ}{c}}_{k↑}^{†}$ and ${\overset{ˆ}{c}}_{-k↓}^{†}$ are the creation operator for an electron with spin up and down, respectively, and $u_{k}$ and $v_{k}$ are variational parameters

(64) $$u_{k}=\cos\left(\frac{\theta_{k}}{2}\right)$$

(65) $$v_{k}=e^{i\phi }\sin\left(\frac{\theta_{k}}{2}\right),$$

to be defined by minimising the total free energy of the system to explain the superconducting phase transition [54], [56]. Here, what is important is the fact that all Cooper pairs occupy the state with zero momentum, ħk−ħk=0. In other words, the Cooper pairs, made of 2 Fermions for each, are occupying the same state with zero momentum among many other choices with arbitrary finite momentum. In that respect, the Cooper pairs exhibit Bose-Einstein condensation. Moreover, the U(1) phase degree of freedom is spontaneously broken, which is evident by the fixed phase of *ϕ*. Here, it is very important that *ϕ* has no dependence on **k** and the phase of *ϕ* is common for all Cooper pairs. The phase coherence is allowed because of the fluctuation of the number of Cooper pairs, since the BCS state enables the superposition state of a different number of pairs.

To see the similarity to our wavefunction of |θ,ϕ〉 for photons, we define the creation operator of a Cooper pair, ${\overset{ˆ}{b}}_{k}^{†}={\overset{ˆ}{c}}_{k↑}^{†}{\overset{ˆ}{c}}_{-k↓}^{†}$, and re-write the BCS wavefunction as

(66) $$|\phi \rangle =\prod_{k}u_{k}\left[1+\frac{v_{k}}{u_{k}}{\overset{ˆ}{c}}_{k↑}^{†}{\overset{ˆ}{c}}_{-k↓}^{†}\right]|0\rangle$$

(67) $$\propto \prod_{k}\left[1+e^{i\phi }\tan\left(\frac{\theta_{k}}{2}\right){\overset{ˆ}{b}}_{k}^{†}\right]|0\rangle$$

(68) $$=\prod_{k}\exp\left[e^{i\phi }\tan\left(\frac{\theta_{k}}{2}\right){\overset{ˆ}{b}}_{k}^{†}\right]|0\rangle ,$$

since ${\overset{ˆ}{b}}_{k}^{n}=0$ for all integers of n≥2. The comparison of the theory of superconductivity with the SU(2) theory for photons described in this paper is summarised in Table 1. Here, we discussed a simple superconducting order parameter of Δ with *s*-wave symmetry, but it can contain internal structures, characterised by orbitals like *p*-wave [98] for ^3^He and *d*-waves for cuprates [99], [100]. The same is true for our photonic state, and the confinement order parameter of $\Delta =\Delta_{n}^{m}$ is indeed characterised by the orbital angular momentum of *m* and the radial quantum number of *n*.

**Table 1:** Comparison of macroscopic coherent states in a laser source and superconductivity. SU(2) symmetry of polarisation state is broken upon lasing, described by the Stokes parameters as expectation values of spin of photons. Coherent state of photons is described by the Bose-Einstein condensation of photons to occupy the single mode described by a spinor wavefunction with fixed polarisation state. The theory of superconductivity is explained by the Bose-Einstein condensation of Cooper pairs to zero momentum state for the centre-of-gravity motion.

System	Symmetry	Phases	Order parameters	Wavefunction
Laser	SU(2)	*θ* and *ϕ*	$S=\langle \overset{ˆ}{S}\rangle$	$e^{\sqrt{N}e^{-i\frac{\phi }{2}}\cos\left(\frac{\theta }{2}\right){\overset{ˆ}{a}}_{L}^{†}}e^{\sqrt{N}e^{+i\frac{\phi }{2}}\sin\left(\frac{\theta }{2}\right){\overset{ˆ}{a}}_{R}^{†}}|0\rangle$
Superconductivity	*U*(1)	*ϕ*	Δ	$\prod_{k}e^{e^{i\phi }\tan\left(\frac{\theta_{k}}{2}\right){\overset{ˆ}{b}}_{k}^{†}}|0\rangle$

### 3.3 Why the SU(2) symmetry is appropriate for a photon with spin 1?

We would like to address the grand challenge of explaining why the SU(2) symmetry is appropriate for a photon with spin 1 [30]. We have considered the GRIN fibre to employ a mathematically rigorous treatment and found that the SU(2) symmetry reasonably appeared from the 2*D* Dirac equation through the relativistic covariant requirement to enable factorisation. The 2×2 Pauli matrices arose to satisfy the anti-commutation relationship, and we have also confirmed that the rotational symmetry of the spin state is guaranteed by considering the *x*, *y*, and *z* components of the spin. Therefore, we could at least show that the existence of the SU(2) symmetry as an internal spin degree of freedom is required to describe the propagation of a photon in a GRIN fibre. This means that a photon has two degrees of freedom during propagation. This corresponds to the fact that the electro-magnetic wave is a transverse wave with two orthogonal directions for electro-magnetic oscillations, perpendicular to the direction of propagation [101][11][12][9]. The most essential part of this derivation is that the photon is effectively massive in the fibre with the finite energy gap of Δ [53][54][56][57][50][51][52].

On the other hand, the present theory of 2*D* Dirac equation is not satisfactory enough to account for the magnitude of the spin of a photon. The naive expectation of spin 1/2 is experimentally denied, and it is well-established that a photon has spin 1 [1][2][42][43][7][8][9][44]. To theoretically derive the magnitude of the spin angular momentum of a photon, we have used the correspondence to classical mechanics. It is well-known that the Poynting vector of $\overset{ˆ}{p}$ corresponds to momentum, and thus the angular momentum density operator is defined as $\overset{ˆ}{m}=r\times \overset{ˆ}{p}$ [46][32]. After integrating over space, we obtain the spin angular momentum operator of ${\overset{ˆ}{S}}_{z}=\hbar \left({\overset{ˆ}{n}}_{L}-{\overset{ˆ}{n}}_{R}\right)$, but ${\overset{ˆ}{S}}_{x}$ and ${\overset{ˆ}{S}}_{y}$ vanish [30][46][32]. This was a well-known paradox, but we believe it is appropriate to obtain only the chiral component of ${\overset{ˆ}{S}}_{z}$ from the classical correspondence, since ${\overset{ˆ}{S}}_{z}$ stands for Ising spin. Classical-mechanics does not possess the concept of the quantum-mechanical superposition state to describe the spin state, whose spin is pointing towards the *x* and *y* directions. Nevertheless, the classical correspondence was a powerful prescription to determine the appropriate expression for ${\overset{ˆ}{S}}_{z}$, which indicates a photon has spin 1.

These two facts, (1) a photon has the SU(2) symmetry and (2) the chiral spin component is ${\overset{ˆ}{S}}_{z}$, are sufficient to obtain the remaining components of ${\overset{ˆ}{S}}_{x}=\hbar \left({\overset{ˆ}{a}}_{L}^{†}{\overset{ˆ}{a}}_{R}+{\overset{ˆ}{a}}_{R}^{†}{\overset{ˆ}{a}}_{L}\right)$ and ${\overset{ˆ}{S}}_{y}=\hbar \left(-i{\overset{ˆ}{a}}_{L}^{†}{\overset{ˆ}{a}}_{R}+i{\overset{ˆ}{a}}_{R}^{†}{\overset{ˆ}{a}}_{L}\right)$, simply by standard SU(2) operations to rotate from ${\overset{ˆ}{S}}_{z}$ [46][32]. Consequently, the commutation relationship for photons becomes

(69) $$[{\overset{ˆ}{S}}_{\alpha },{\overset{ˆ}{S}}_{\beta }]=i2\hbar \epsilon_{\alpha \beta \gamma }{\overset{ˆ}{S}}_{\gamma },$$

where $\epsilon_{\alpha \beta \gamma }$ is a complete anti-symmetric tensor and *α*, *β*, *γ* = *x*, *y*, *z*. The right-hand side is a factor of 2 larger than the commutation relationship for spin 1/2, which means that we only need to transfer ħ/2→ħ to convert the commutation relationship from spin 1/2 to spin 1. This is consistent with the fact that a photon has only two internal degrees of freedom, while the magnitude of the spin angular momentum is *ħ* as spin 1.

### 3.4 Extension of the theory from a GRIN fibre to free space

In this work, we have restricted our analysis to a GRIN fibre, such that our results are valid strictly only in the system. However, we will discuss briefly the validity and the possible break down of the theory in free space or a vacuum. In the present theory, we have only one parameter, *g*, to account for the spatial profile. The free space limit corresponds to g→0, describing a uniform material with the refractive index of $n_{0}$. This corresponds to Δ→0, meaning that the confinement would be weaker and the effective mass vanishes. Suppose *g* is decreasing as the photon propagates along *z*, the mode gradually expands to the *x* and *y* directions, and a photon distributes to the entire space. A photon is an elementary particle, and thus its distribution must be described by a wavefunction [42][43][7][8][40] in quantum mechanics. In the absence of confinement at g=0, a photon has no preferential direction for propagation, making it difficult to assign the directions of oscillations perpendicular to the propagation direction. Therefore, it is challenging to apply our theory of the 2*D* Dirac equation to the free space. In fact, it is in this free space limit where many issues arise in splitting angular momentum into spin and orbital angular momentum [23][24][25][28][29][31]. Historically, quantum mechanics was developed to account for blackbody radiation to measure the temperature of a blast furnace and to refine ion production [42][43]. In a blackbody, photons spread throughout the entire system, and existing in the superposition states among various propagation directions. Our theory is not applicable in this limit.

Nevertheless, in most laser optic experiments, the laser beam is sufficiently collimated to account for the direction of propagation. As far as the confinement is finite in optical fibres, we expect a finite dispersion relationship between energy and momentum along the direction of propagation. In these cases, we expect that optical confinement induces finite effective mass for a photon propagating in the fibre, enabling us to apply our theory to understand the origin of polarisation from the intrinsic spin of a photon. If the mode profile is finite, we have shown that angular momentum can be split into spin and orbital angular momentum in a gauge invariant way [32]. In broken symmetric fibres such as polarisation-maintain fibres [11], the polarisation state would be aligned to some preferential direction, which can be described by the SU(2) wavefunction, known as Jones vector [101][11][12][13]. We hope that we have provided a theoretical foundation to consider the polarisation state by the SU(2) wavefunction. Even in free space or a vacuum, we consider the spin angular momentum operators $\left({\overset{ˆ}{S}}_{x},{\overset{ˆ}{S}}_{y},{\overset{ˆ}{S}}_{z}\right)$ are useful as long as the beam is collimated [32]. If we accept the definition of the obtained spin angular momentum operators, the Stokes parameters on the Poincaré sphere can be interpreted as the spin expectation values by photons in a coherent state [46][32].

## 4 Application to topological polarisation state

We have discussed the origin of polarisation from the Dirac equation for photons in a waveguide. The relativistic covariant requirement induced two mutually orthogonal states to describe an SU(2) spin state of a photon. Due to the rotational symmetry of the waveguide, the spin of a photon can point in any direction, which is described by the superposition state of two orthogonal basis states of SU(2). For coherent photons emitted from a laser source, the many-body state is described by a coherent state, which also possesses the SU(2) degree of freedom due to the nature of Bose-Einstein statistics. We have also confirmed that the Stokes parameters are actually spin expectation values of coherent photons [76][77][11][12][13][46][102], and the radius of the Poincaré sphere ($S_{0}$) depends on the power intensity of the light source.

However, one could argue that most of these results we have obtained so far are well-known [101][11][12][13] and thus trivial. In fact, two orthogonal states of photons simply mean that an electromagnetic wave propagating in a waveguide is a transverse wave, such that only two components out of the three spatial components are observable for a photon, which has an intrinsic spin of 1 [11][7][8][46]. There have been numerous attempts to derive the optical Dirac equation [17][18][19][20][36][37][38][39][40] [41][87][88][71], and we have not demonstrated sufficiently novel results that could be confirmed in new experiments.

Here, we will discuss a topological aspect of polarisation states [102], as an application of our considerations. In particular, we focus on the difference between the SU(2) wavefunction of spin states and SO(3) expectation values. Mathematically, the fundamental theorem of homomorphism [103][104][105][106][107] gives their relationship as SU(2)/$S^{0}≅$SO(3), where $S^{0}=\{-1,1\}$. This means that the phase change of −1 for the SU(2) wavefunction is expected upon a 2*π* rotation on the Poincaré sphere, described by SO(3). This phase difference is observable in the interference experiments. We show that an energy band structure similar to a Dirac cone [108][109][110][111][112][113] is expected.

### 4.1 Polarisation interferometer

**Figure 1:**
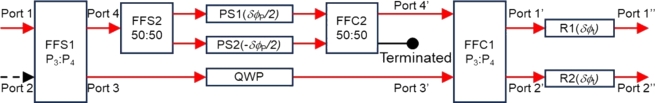
Polarisation interferometer. The diagonally polarised input beam is inserted from Port 1, and Port 2 is not used. The beam is split by FFS1 at the power splitting ratio of *P*_3_:*P*_4_ into Ports 3 and 4, respectively. The beam from Port 3 is passing through the QWP to be the left-circularly polarised state, while the intensity of the beam from Port 4 was controlled by a Mach-Zehnder interferometer in the push-pull configuration. The beams are combined by FFC1 and subsequently rotated by rotators, R1 and R2. Abbreviations are as follows: FFS, Fibre-to-Fibre Splitter; FFC, Fibre-to-Fibre Coupler; PS, Phase-Shifter; R, Rotator; QWP, Quarter-Wave-Plate.

To see the change of intensities upon interference, we propose the polarisation interferometer, as shown in Fig. 1. The central idea is to split a beam into two beams, among which one beam is fixed to a certain polarisation orientation, while the phase and the amplitude of the other beam are controlled by the SU(2) operation. The split beams are then recombined to see the interference.

The example of the set-up, shown in Fig. 1, assumes the input of the diagonally polarised beam, which is inserted into the Fibre-to-Fibre-Splitter (FFS) at the power splitting ratio of $P_{3}:P_{4}$ into Port 3 and Port 4, respectively. We have not considered using input Port 2. The beam from Port 3 is passing through the Quarter-Wave-Plate (QWP), whose fast axis is aligned to the horizontal direction, and we expect the beam to be in a left-circularly polarised state, as seen from the detector side. The beam is subsequently inserted into Port 3' of the Fibre-to-Fibre-Combiner (FFC1) and becomes the reference polarisation state to interfere with the input from Port 4'.

The other beam out of Port 4 is inserted into a Mach-Zehnder (MZ) interferometer, made of FFS2 and FFC, connected by Phase-Shifters (PS1 and PS2) in the push-pull configuration to change the phases of the beams in the upper and lower arms by amounts of $\delta \phi_{p}/2$ and $-\delta \phi_{p}/2$, respectively. This enables control of the amplitude and phase for the beam entering Port 4'.

After the interference at FFC1, the outputs from Port1' and Port2' are controlled by rotators (R1 and R2) to change the polarisation state in the $S_{1}$-$S_{2}$ plane, and the final outputs are observed by Port 1” and Port 2” with an amount of $\delta \phi_{t}$.

### 4.2 Numerical calculations

We numerically calculated SU(2) wavefunctions and spin expectation values. The output power linearly depends on the input power, such that $S_{0}=\hbar N$ of the output also depends on the input. Therefore, we have assumed that the output of the Stokes parameters is normalised by the input intensity of $S_{0}$. We can also consider assuming that the Stokes parameters essentially have a unit of power, such that if the input is 1 mW, the calculated Stokes parameters also have the unit of mW. $S_{0}$ essentially corresponds to the energy of the many-body coherent state.

**Figure 2:**
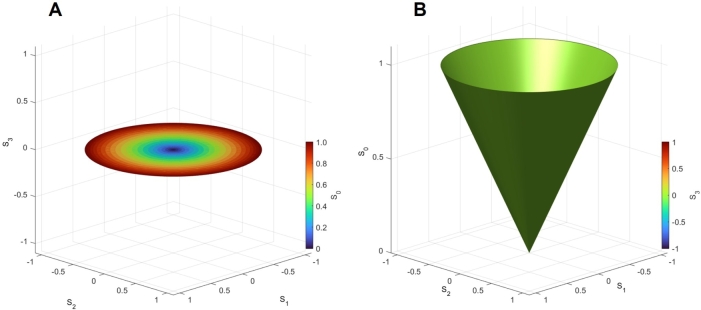
Impact of Mach-Zehnder interferometer on the output state. We have assumed the splitting of *P*_3_:*P*_4_ = 0:100 and calculated the Stokes parameters of the output beam at Port 1”. We have scanned to change both *δϕ*_p_ and *δϕ*_t_ from 0 to 2*π*. (A) Spin expectation values of (*S*_1_,*S*_2_,*S*_3_). Trajectories of *S*_1_ and *S*_2_ have 2*D* rotational symmetry, while the chiral component of *S*_3_ is always zero. (B) Energy dispersion of (*S*_1_,*S*_2_,*S*_0_). The radius of the circle, $\sqrt{S_{1}^{2}+S_{2}^{2}}$, corresponds to *S*_0_.

First, we confirmed the impact of the MZ interferometer by assuming the splitting of $P_{3}:P_{4}=0:100$, as shown in Fig. 7. We confirmed that the 2*D* rotational symmetry of the trajectories in the Stokes parameters space of $\left(S_{1},S_{2},S_{3}\right)$, which comes from the rotator operations to change $\delta \phi_{t}$ from 0 to 2*π* (Fig. 2A). Contrary to the standard description on the Poincaré sphere, we discuss the change of intensity upon interference, such that the trajectories become a disk (Fig. 2), instead of the equator on the Poincaré sphere. Please note that the chiral component of $S_{3}$ vanishes, since the interference merely changes the intensity, and the rotator operation does not induce the chiral component. The intensity of the beam ($S_{0}$) is controlled from 0 to 1 by PSs to change $\delta \phi_{p}$ from 0 to 2*π* (Fig. 2B), and it increases linearly upon increasing the radius of the disk.

In the opposite limit of the splitting at $P_{3}:P_{4}=100:0$, all the intensity is going through Port 3, and the output appears from Port 1', whose Stokes parameters are not affected by R1. In this case, the output from Port 1” becomes $S_{0}=S_{3}=1$ and $S_{1}=S_{2}=0$ (which is only a point and not shown). The corresponding left-circularly polarised state becomes a reference state to see the interference with the beam from Port 4'.

**Figure 3:**
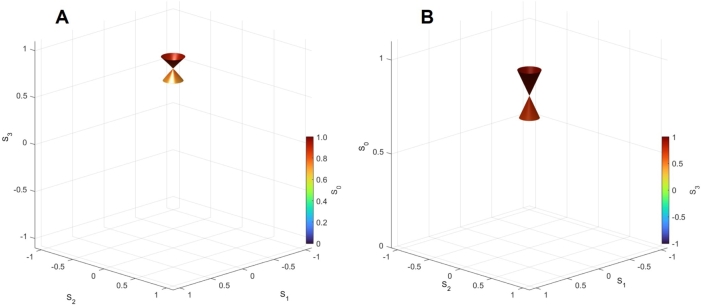
Dirac Bosons calculated at the splitting of *P*_3_:*P*_4_ = 90:10 for the output beam at Port 1”. We have scanned to change both *δϕ*_p_ and *δϕ*_t_ from 0 to 2*π*. (A) Spin expectation values of (*S*_1_,*S*_2_,*S*_3_). The Dirac point is found at (0,0,*S*_D_)=(0,0,0.81). (B) Energy dispersion of (*S*_1_,*S*_2_,*S*_0_).

Next, we calculated the Stokes parameters at $P_{3}:P_{4}=90:10$ (Fig. 3) and found that $S_{0}$ and $S_{3}$ are affected by the interference between beams from Port 3' and Port4'. Both positive and negative interferences are taking place upon changing $\delta \phi_{p}$. We found the Dirac point located at $\left(S_{1},S_{2},S_{3})=(0,0,S_{D})=(0,0,0.81\right)$, where the polarisation state is not changed upon the rotator operation. We confirmed that the energy dispersion is linear against $S_{1}$ and $S_{2}$, reminiscent of a Dirac cone found in Graphene [108][109][110][111][112][113]. However, there are quite a few differences between the present Dirac Boson and the well-established Dirac Fermion [108][109][110][111][112][113]. The main difference is the quantum statistics of an elementary particle, involved. For Graphene, the quasi-particle dispersion in a honeycomb lattice is discussed, such that the energy is for the single particle, which is a Fermion. On the other hand, we are focusing on the energy of many-body states for coherent photons, which are Bosons. The physical parameter for the energy dispersion is different. For Graphene, the 2D momentum of $k=(k_{1},k_{2})$ is changed from the K$=2\pi /a(1/3,1/\sqrt{3})$ and K'=2π/a(2/3,0) points in the band structure with the lattice constant of *a*, while we confirmed $S_{0}$ is linear against $S_{1}$ and $S_{2}$ at the Dirac point of $\left(0,0,S_{D}\right)$.

**Figure 4:**
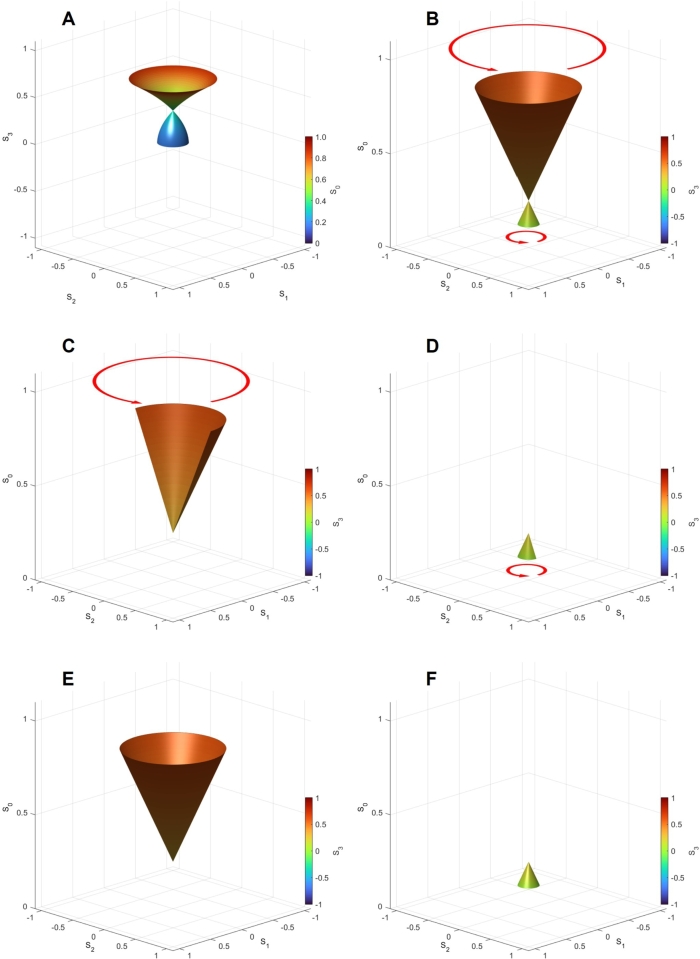
Dirac Bosons calculated at the splitting of *P*_3_:*P*_4_ = 50:50. (A) Spin expectation values of (*S*_1_,*S*_2_,*S*_3_) and (B) Energy dispersion of (*S*_1_,*S*_2_,*S*_0_) for the output beam at Port 1”. We have scanned to change both *δϕ*_p_ and *δϕ*_t_ from 0 to 2*π*. The direction of the arrow is shown to describe the left rotation, induced by changing *δϕ*_t_ from 0 to 2*π*. Energy dispersion of (*S*_1_,*S*_2_,*S*_0_) for (C) Port 1” and (D) Port 2”, calculated by changing *δϕ*_p_ from 0 to *π* and changing *δϕ*_t_ from 0 to *π*, respectively. The change in *δϕ*_t_ induces the left circulation of (*S*_1_,*S*_2_) along *S*_3_ for both ports, while the larger intensity is found in Port 1” compared with the lower intensity in Port 2”. We have also calculated energy dispersion of (*S*_1_,*S*_2_,*S*_0_) from Port 1” by changing *δϕ*_p_ (E) from 0 to *π* and (F) from *π* to 2*π*, respectively. The constructive interference is achieved for (E), while the destructive interference reduces the intensity for (F). The Dirac point is found at (0,0,*S*_D_)=(0,0,0.25).

We have also calculated the Stokes parameters at the splitting of $P_{3}:P_{4}=50:50$ to see the impacts of various contributions. At this splitting, the Dirac point is located at $\left(0,0,S_{D})=(0,0,0.25\right)$ (Fig. 4A), and such that the lower band below $S_{D}$ ($S_{0}<S_{D}$) has almost vanished due to the destructive interference, while the upper band ($S_{0}>S_{D}$) is larger due to the constructive interference (Fig. 4B). This splitting condition is close to the condition to have a high extinction ratio in a MZ interferometer. The parameters of $\left(S_{1},S_{2}\right)$ were rotated along the $S_{3}$ axis towards the left (anti-clock-wise) direction by increasing $\delta \phi_{t}$ from 0 to 2*π*. We have also compared the output from Port 1” (Fig. 4C) with that from Port 2” (Fig. 4D), which shows the output intensity is complementary; if one port is constructive, the other port is destructive in interference. The directions of rotations are the same, since they are simply rotated by R1 and R2 after the interference. We have also checked the impact of $\delta \phi_{p}$ to achieve the constructive interference (Fig. 4E) and the destructive interference (Fig. 4D).

**Figure 5:**
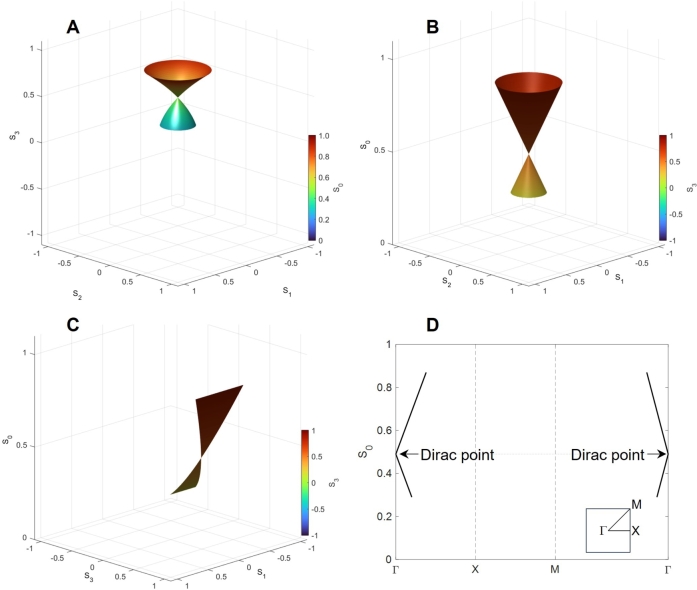
Dirac Bosons calculated at the splitting of *P*_3_:*P*_4_ = 70:30. (A) Spin expectation values of (*S*_1_,*S*_2_,*S*_3_), (B) Energy dispersion of (*S*_1_,*S*_2_,*S*_0_), and (C) Energy dispersion of (*S*_1_,*S*_3_,*S*_0_) for the output beam at Port 1”. The Dirac point is found at (0,0,*S*_D_)=(0,0,0.49). We have scanned to change both *δϕ*_p_ and *δϕ*_t_ from 0 to 2*π*. (D) 2*D* band structure to change parameters in the (*S*_1_,*S*_2_) plane.

Then, we also calculated the Stokes parameters at the splitting of $P_{3}:P_{4}=70:30$ to see the extended lower band (Fig. 5A and 5B). In this case, the Dirac point is found at $\left(0,0,S_{D})=(0,0,0.49\right)$, which is located almost at the middle of the normalised $S_{0}$ axis. We also confirmed the linearity of $S_{0}$ against $S_{3}$ near the Dirac point (Fig. 4C). The energy dispersion of $S_{0}$ against $S_{1}$ and $S_{2}$ can also be shown as a 2*D* band structure [108][109] (Fig. 4D). Here, we followed the convention for describing the band structure in 2D [108][109], but our parameters are the normalised spin expectation value of $\left(S_{1},S_{2}\right)$ instead of the momentum of $k=(k_{1},k_{2})$. We defined the Γ point at $\left(S_{1},S_{2})=(0,0\right)$, the X point at (1,0), and the M point at (1,1). Note that we could not cover the complete 2*D* surface under our calculation conditions upon changing $\delta \phi_{p}$ and $\delta \phi_{t}$. Therefore, the allowed state is restricted to $\left(S_{1},S_{2}\right)$ as well as $S_{0}$. In this sense, the energy band can only cover the restricted space in $\left(S_{1},S_{2},S_{3}\right)$, as described in Fig. 4A. For the coherent photons, we have an identity of $S_{0}=\sqrt{S_{1}^{2}+S_{2}^{2}+S_{3}^{2}}$, such that the value of $S_{3}$ is fixed once $\left(S_{1},S_{2},S_{0}\right)$ is fixed.

**Figure 6:**
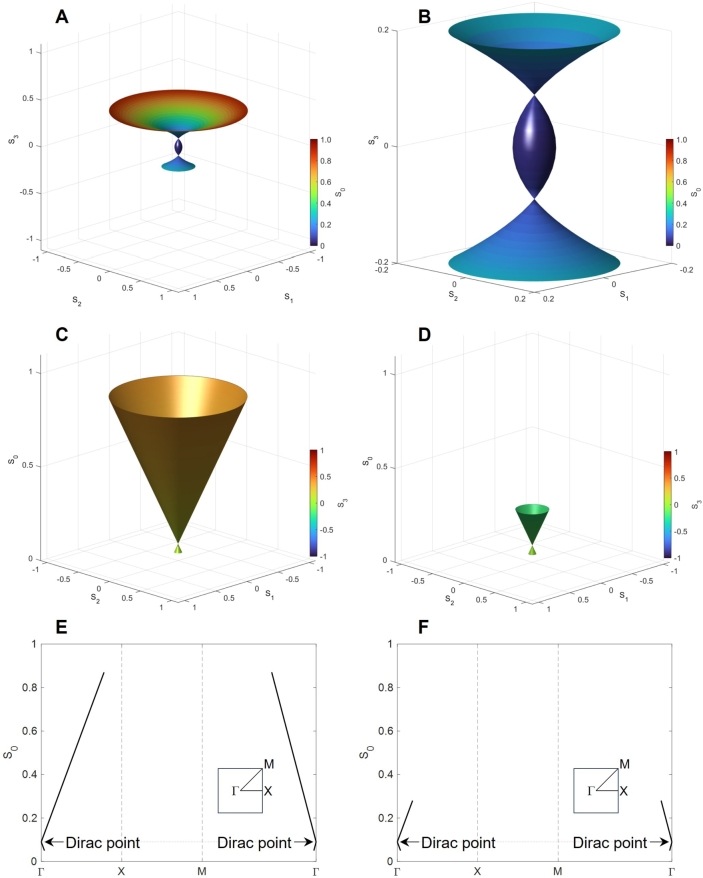
Dirac Bosons calculated at the splitting of *P*_3_:*P*_4_ = 30:70. (A) Spin expectation values of (*S*_1_,*S*_2_,*S*_3_) and (B) its expanded image, calculated by changing both *δϕ*_p_ and *δϕ*_t_ from 0 to 2*π*. (C) Dirac cone with the upper Dirac point of (0,0,*S*_D_) and (D) Dirac cone with the lower Dirac point of (0,0,−*S*_D_) at *S*_D_ = 0.09. 2*D* energy band diagram of (E) upper band and (F) lower band in the (*S*_1_,*S*_2_) plane.

Finally, we calculated the opposite splitting of $P_{3}:P_{4}=30:70$, as shown in Fig. 6. In this case, the beam of the larger intensity is inserted into Port 4, compared with Port 3, such that the contribution of the larger intensity is affected by the phase change of $\delta \phi_{p}$. Consequently, we found two Dirac points at $\left(0,0,S_{D}\right)$ and $\left(0,0,-S_{D}\right)$ with the parameter of $S_{D}=0.09$ (Fig. 6A and 6B). In this parameter range, the upper Dirac cone is even larger (Fig. 6C), while we also confirmed the presence of the lower Dirac cone located at the negative $S_{3}$ (Fig. 6D). Note that we plotted the $S_{0}$ axis pointing upwards in Fig. 6D, while the values of $S_{3}$ are negative for the lower Dirac cone, such that the axis of $S_{3}$ in Fig. 6B is opposite for the lower Dirac cone $\left(S_{0}>0,S_{3}<0\right)$. We also showed the 2*D* band diagrams for both Dirac cones, in Figs. 6E and 6F, respectively. The Dirac point is located at the same $S_{0}=S_{D}=0.09$, such that the energy levels are degenerate near the Dirac point. On the other hand, the upper Dirac cone is extended more over the $\left(S_{1},S_{2}\right)$ plane (Fig. 6E), compared with the lower Dirac cone (Fig. 6F).

### 4.3 Effective Hamiltonian for spin expectation values in free space

We will describe the effective Hamiltonian for the topological Dirac cone emerged from the device, named the polarisation interferometer. But, before considering our specific example in proposed polarisation interferometers, it would be useful to consider it in free space.

We begin our discussions by reviewing how the band structure of Graphene is formed, as a successful example to produce Dirac Fermions [108][109]. In free space, the Schrödinger equation for an electron simply produces parabolic dispersion, determined by the vacuum mass of an electron, due to the continuous translational symmetry [114]. The energy of the continuous surface gives the Fermi surface, which is a sphere of $S^{2}$ for 3*D* space. Then, we consider the energy dispersion in a 2*D* Graphene [108][109]. The continuous translational symmetry is broken due to the honeycomb lattice structure, and electrons are tightly trapped in carbon atoms and quantum tunnelling is taking place to move among atoms. If we employ this tight-binding model for electrons in Graphene, it is useful to consider an AB bipartite lattice, and it is this sublattice degree of freedom that is responsible for SU(2) states [108][109].

Similarly, we would like to consider an effective Hamiltonian to describe coherent photons. Here, the energy is not a single-particle energy for a photon, and we consider the many-body energy for coherent photons. Nevertheless, due to Bose-Einstein statistics and the coherence of photons, we only need to consider the energy of $E_{0}=\omega S_{0}$ under the SU(2) symmetry for spin states. In consideration of the energy for the spin of photons, it would be useful to consider a conjugate variable to spin, which would be a hypothetical magnetic field of $h=(h_{1},h_{2},h_{3})$. This cannot be the real magnetic field, since photons do not have charge, and the spin of photons cannot be directly coupled to the real magnetic field. Nevertheless, if we proceed to assume a similarity to the Dirac equation for an electron, we obtain a photonic Dirac equation as

(70) $$i\hbar \frac{\partial }{\partial t}\Psi =\omega \left(\sigma_{1}\frac{\partial }{\partial h_{1}}+\sigma_{2}\frac{\partial }{\partial h_{2}}+\sigma_{3}\frac{\partial }{\partial h_{3}}\right)\Psi$$

(71) $$\equiv \omega \sigma \cdot \nabla_{h}\Psi ,$$

where *Ψ* is a many-body wavefunction for coherent photons. Considering the Stokes parameters are conserved in free space, we assume

(72) $$\Psi =e^{-\frac{i}{\hbar }\omega S_{0}t}e^{iS_{1}h_{1}+iS_{2}h_{2}+iS_{3}h_{3}},$$

which yields the Hamiltonian

(73) $$\overset{ˆ}{H}=\omega \left(\begin{array}{cc} S_{3}\; & S_{1}-iS_{2}\\ S_{1}+iS_{2}\; & -S_{3} \end{array}\right).$$

By diagonalising $\overset{ˆ}{H}$, we obtain $S_{0}=\pm \sqrt{S_{1}^{2}+S_{2}^{2}+S_{3}^{2}}$. By taking the positive value for $S_{0}$, we obtain $S_{0}=\sqrt{S_{1}^{2}+S_{2}^{2}+S_{3}^{2}}$ for free space. Regardless of the unusual description of the many-body coherent state, it is surprising to get the correct form for the polarisation state [76][77][11][12][13][46][102], which provides the rotationally symmetric feature in the form of a Poincaré sphere.

### 4.4 Dirac equation for polarisation interferometer

Finally, we discuss the effective Hamiltonian for our polarisation interferometer to describe the Dirac Bosons. In the proposed polarisation interferometer (Fig. 1), the chiral symmetry is broken, such that the parameter $S_{3}$ is changed upon constructive or destructive interference by PS1 and PS2. On the other hand, 2*D* rotational symmetry is still maintained by R1 and R2. Consequently, we assume a 2*D* photonic Dirac equation as

(74) $$i\hbar \frac{\partial }{\partial t}\Psi =\omega \left(\sigma_{1}\frac{\partial }{\partial h_{1}}+\sigma_{2}\frac{\partial }{\partial h_{2}}\right)\Psi ,$$

whose solution would be

(75) $$\Psi =e^{-\frac{i}{\hbar }\omega (S_{0}-S_{D})t}e^{iS_{1}h_{1}+iS_{2}h_{2}},$$

which yields the Hamiltonian

(76) $$\overset{ˆ}{H}=\left(\begin{array}{cc} \pm S_{D}\; & S_{1}-iS_{2}\\ S_{1}+iS_{2}\; & \pm S_{D} \end{array}\right),$$

and the dispersion relationship

(77) $$S_{0}=S_{D}\pm \sqrt{S_{1}^{2}+S_{2}^{2}}.$$

Consequently, the 2D Dirac equation provides Bosonic Dirac cones. We have confirmed this simple dispersion is in complete agreement with the numerical calculations of Figs. 5A, 5E, and 5F. The summary of the symmetry consideration is shown in Table 2.

**Table 2:** Summary of Dirac cones for various systems.

	Graphene	Polarisation interferometer
		**Table:** *P*_3_:*P*_4_ = 70:30 *P*_3_:*P*_4_ = 30:70
Elementary particles	Electrons	Photons
SU(2) states	AB sublattices	Spin=Polarisation
Broken symmetry	Translational symmetry	Chirality
Preserved symmetry	Honeycomb lattice	Rotational SO(2) symmetry
Number of valleys	2	**Table:** 1 2
Dirac points	K, K'	**Table:** (0,0,*S*_D_) (0,0,±*S*_D_)
Hamiltonian	$\left(\begin{array}{cc} 0\; & k_{1}\mp ik_{2}\\ k_{1}\pm ik_{2}\; & 0 \end{array}\right)$	**Table:** $\left(\begin{array}{cc} S_{D}\; & S_{1}-iS_{2}\\ S_{1}+iS_{2}\; & S_{D} \end{array}\right)$ $\left(\begin{array}{cc} \pm S_{D}\; & S_{1}-iS_{2}\\ S_{1}+iS_{2}\; & \pm S_{D} \end{array}\right)$

## 5 Conclusion

Spin is an inherent degree of freedom to characterise the polarisation state for a photon. We have discussed the wavefunction for a photon described by a Helmholtz equation in a graded-index fibre, which can be solved exactly using special functions such as Laguerre-Gauss and Hermite-Gauss functions. The energy spectrum is described by the massive Schrödinger equation, which could be transferred to the Klein-Gordon equation by a simple unitary transformation for shifting the energy due to the condensation for lasing, accompanied by the confinement in the fibre. The two-dimensional Klein-Gordon equation is factorised to be the Dirac equation for a photon enabling the quantum-mechanical probabilistic interpretation. The decoupled Dirac equation has exactly the same form as the spin equation of motion under the effective magnetic field. We have shown the full spherical symmetry of the spin state by the rotational symmetry of spin using SU(2) operators. The theory of spin state for a photon has a close similarity with the theory of superconductivity due to the intrinsic 2-level nature of polarisation state and pairing state. We have shown that the macroscopic quantum coherent state is a manifestation of the Bose-Einstein condensation nature of the coherent state described by the broken SU(2) symmetry of the polarisation state. Consequently, the spin expectation values calculated by the coherent state become the Stokes parameters on the Poincaré sphere.

As an application of our theory, we have proposed a polarisation interferometer, which breaks the chiral symmetry of the polarisation states upon interference. We have numerically calculated the Stokes parameters for various splitting ratios of the fibre splitter and found that 2*D* Dirac cones are expected upon changing the Stokes parameters. The effective Hamiltonian of the system is described by the Dirac equation in the Stokes parameter space. We believe many other topological features could be realised as polarisation states upon controlling the SU(2) freedom of coherent photons.

## CRediT authorship contribution statement

**Shinichi Saito:** Writing – review & editing, Writing – original draft, Visualization, Validation, Supervision, Software, Resources, Project administration, Methodology, Investigation, Funding acquisition, Formal analysis, Data curation, Conceptualization.

## Declaration of Competing Interest

The author declares the following financial interests/personal relationships which may be considered as potential competing interests: Shinichi Saito reports financial support was provided by 10.13039/501100001691Japan Society for the Promotion of Science. Shinichi Saito reports financial support was provided by Hitachi Ltd.

## Appendix A Supplementary material

MMC 1 The supplementary information on the photonic Dirac equation is provided. MMC 1

MMC 2 Data1 corresponds to the numerical data of calculated Stokes parameters for Fig. 2. Stokes parameters of S0, S1, S2, and S3, are shown in the corresponding worksheets. S3 against S1 and S2 are shown in Fig. 2 (A). S0 against S1 and S2 are shown in Fig. 2 (B). MMC 2

MMC 3 Data1 corresponds to the numerical data of calculated Stokes parameters for Fig. 3. Stokes parameters of S0, S1, S2, and S3, are shown in the corresponding worksheets. S3 against S1 and S2 are shown in Fig. 3 (A). S0 against S1 and S2 are shown in Fig. 3 (B). MMC 3

MMC 4 Data1 corresponds to the numerical data of calculated Stokes parameters for Fig. 4. Stokes parameters of S0, S1, S2, and S3, are shown in the corresponding worksheets. S3 against S1 and S2 are shown in Fig. 4 (A). S0 against S1 and S2 are shown in Fig. 4 (B). MMC 4

MMC 5 Data1 corresponds to the numerical data of calculated Stokes parameters for Fig. 5. Stokes parameters of S0, S1, S2, and S3, are shown in the corresponding worksheets. S3 against S1 and S2 are shown in Fig. 5 (A). S0 against S1 and S2 are shown in Fig. 5 (B). MMC 5

MMC 6 Data1 corresponds to the numerical data of calculated Stokes parameters for Fig. 6. Stokes parameters of S0, S1, S2, and S3, are shown in the corresponding worksheets. S3 against S1 and S2 are shown in Fig. 6 (A). S0 against S1 and S2 are shown in Fig. 6 (C). MMC 6
