# Supplementary material for: Dirac equation for photons in a fibre: Origin of polarisation
Source: Heliyon. 2024 Mar 21;10(7):e28367. doi: 10.1016/j.heliyon.2024.e28367 (PMC11004707; doi:10.1016/j.heliyon.2024.e28367)
Supplement: MMC 1 — The supplementary information on the photonic Dirac equation is provided. [file mmc1.pdf]

Supplementary Information for 'Dirac equation for photons in a fibre: origin of polarisation'

Shinichi Saito[a]

[a] *Center for Exploratory Research Laboratory, Research & Development Group, Hitachi, Ltd., 1-280 Higashi-Koigakubo, Kokubunji, 185-8601, Tokyo, Japan*

Abstract

The supplementary information on the photonic Dirac equation is provided.

Keywords: Dirac equation, Klein-Gordon equation, Polarisation, Spin Angular Momentum, Coherent state, Broken symmetry, Graded index fibre

1. Introduction

We have recently considered what is spin of a photon by using a standard quantum many-body field theory [1]. We assumed that a coherent ray of photons emitted from a laser source, described by a coherent state with macroscopic number of photons in a waveguide, and showed that the quantum-mechanical spin operators, $\hat{\mathbf{S}}$, are derived by using an SU(2) Lie algebra [1]. Contrary to the popular view that the coherent ray is classical, we showed that the polarisation state is described by the quantum-mechanical average of spin operators, $\langle \hat{\mathbf{S}} \rangle$, because of the Bose-Einstein condensation of photons [1]. The phase and the amplitude of the macroscopic wavefunction can be easily controlled by passive optical components such as a phase-shifter and a rotator [2, 3, 4, 5, 6, 7], while the SU(2) group theory is a powerful tool to describe the spin state [8, 9, 10, 2, 11, 12, 2, 13, 14, 15, 16, 17, 18, 19, 20, 1]. It was shown that the spin expectation value, $\langle \hat{\mathbf{S}} \rangle$, is exactly the same as Stokes parameters (S_1, S_2, S_3) on the Poincaré sphere [21, 22, 1]. Therefore, we believe that spin of a photon is well-defined quantum-mechanically and spin is an inherent property of a photon as an elementary particle.

We have also analysed the commutation relationship of orbital angular momentum operators for a photon [23, 24], described by a Laguerre-Gauss mode [25, 2]. We showed that the ladder operators to increment and decrement the

orbital angular momentum along the direction of the propagation in the unit of the Dirac constant, $\hbar = h/(2\pi)$, where h is the Plank constant [23]. We have also shown the validity of the quantum commutation relationship against the Laguerre-Gauss mode, and the Laguerre-Gauss mode is labelled by a definite integer quantum number (m) to describe the magnetic orbital angular momentum of $\hbar m$. The Laguerre-Gauss mode is not an eigenstate for the magnitude of the orbital angular momentum, $\hat{\mathbf{l}} \cdot \hat{\mathbf{l}}$, but nevertheless, the quantum expectation value, $\langle \hat{\mathbf{l}} \cdot \hat{\mathbf{l}} \rangle$, was analytically calculated, which contains both intrinsic and extrinsic contributions [23]. By incrementing m by a ladder operation by using a vortex lens, the magnitude of the orbital angular momentum is increased, which is shown on the nested Poincaré spheres [23, 24]. Based on these results, it was suggested that the orbital angular momentum is also a well-defined physical observable for a photon.

Then, we have revisited the issue of the proper splitting [26] of the total photonic orbital angular momentum into spin and orbital angular momentum [25, 27, 28, 29, 30, 2, 3, 31, 32, 28, 30, 33, 34, 35, 26] . One might think that it is a trivial task to split spin and orbital angular momentum, since there are so many papers, published to manipulate spin and orbital angular momentum, independently [29]. There seems to be no doubt that these degrees of freedom are experimental observables, and it is widely described even in textbooks, e.g. the section 11.3.2 of the reference [36] and the problem 7.27 of the reference [3], that the splitting is achievable. However, one of the most critical step for the derivation is the integration by parts and the dropping of the term from the boundary condition [34][33][29][35][26]. The difficulties and challenges of the splitting were reviewed in the reference [29]. It was considered to be impossible to achieve the splitting [25, 27, 28, 29, 30, 2, 3, 31, 32, 28, 29, 30, 33] without imposing special boundary conditions [33, 29] in a proper gauge invariant way, except for limited numbers of researchers, who believe it possible [34, 35, 26]. We have recently considered the propagation of the Laguerre-Gauss mode in a GRaded-INdex (GRIN) fibre [37] and found that the splitting is indeed possible for confined modes in the fibre [26]. One of the most important part of this derivation was to assume the finite mode size of the wave rather than assuming a simple plane wave expansion. Historically, quantum mechanics was developed by the consideration of the black body radiation [38, 39, 40, 11], for which the plane-wave expansion was appropriate to consider all possible modes in a black body. The field theoretic treatments using creation and annihilation operators, known as the second quantisation techniques, were developed based on the plane wave expansion [41, 12, 42]. However, the plane waves with continuous spectra were not suitable for describing the discrete confined modes with proper symmetries

reflecting the profile of the refractive index in a waveguide. The mode profile is essentially described by a wavefunction of a photon, which is obtained as a solution of the Helmholtz equation [26]. Consequently, there exists the small longitudinal electric field, coming from the finite shape of the envelope wavefunction, which ensures the proper splitting between spin and orbital angular momentum from the total angular momentum [26]. From the classical correspondence of the angular momentum density operator, $\hat{\mathbf{m}} = \mathbf{r} \times \hat{\mathbf{p}}$, between the momentum density operator, $\hat{\mathbf{p}}$, known as the Poynting vector, and the coordinate, $\mathbf{r}$, we obtained the orbital angular momentum operator for spin and orbital angular momentum along the direction of propagation [26]. The projected components of spin and orbital angular momentum operators along the direction of propagation are helicity operators [26]. For the transverse components of the angular momentum, we accepted the principle of the quantum-mechanical rotational symmetries for spin and orbital angular momentum using the SU(2) Lie algebra, which led to the expressions for all components of spin and orbital angular momentum operators [26]. The spin expectation values become Stokes parameters on the Poincaré sphere, as expected, and the expectation values of orbital angular momentum are also described by similar parameters in the higher-order Poincaré sphere [43, 44, 45, 46, 47, 26]. Based on these analyses [43, 44, 45, 35, 1, 23, 26], we are approaching to understand quantum-mechanical features of spin and orbital angular momentum for coherent photons. However, we have not yet established the intrinsic quantum-mechanical origin of spin for a photon, as compared with the level of understanding of spin for an electron using the Dirac equation [48, 41].

2. Results

We discuss the origin of spin for a photon propagating in a GRIN fibre. The interpretation of spin of a photon as a polarisation degree of freedom is discussed.

2.1. Symmetries

Here, we focus on the symmetries of the possible solutions in a GRIN fibre. We go back to the original Helmholtz equation

$$\left[\nabla^2 - \frac{1}{v_0^2}\left(1 - g^2 r^2\right)\partial_t^2\right]\Psi(\mathbf{r}) = 0, \tag{1}$$

and discuss why several solutions appeared. In deriving the dispersion relationship, we realise $\partial_z^2 = (ik)^2 = -k^2$ and $\partial_t^2 = (i\omega)^2 = -\omega^2$, and thus the solutions

are available in dependent of the signs of k and ω_0. Therefore, the wavefunction along the direction of the propagation is expressed as $\psi_z = \mathrm{e}^{\pm ikz \pm i\omega_0 t}$, where 4 possible solutions are available depending on the choice of signs. All solutions are summarised in Table 1. If we consider standard time evolution of $t > 0$, $k > 0$, and $\omega_0 > 0$, the direction of propagation is along the positive z direction ($+z$) for the solutions $\mathrm{e}^{ikz-i\omega_0 t}$ and $\mathrm{e}^{-ikz+i\omega_0 t}$, and it is negative ($-z$) for $\mathrm{e}^{-ikz-i\omega_0 t}$ and $\mathrm{e}^{ikz+i\omega_0 t}$. Alternatively, if we allow k to be both negative and positive, we can always use $\mathrm{e}^{ikz-i\omega_0 t}$, while we should consider 2 branches for each mode. Gapful modes (Suppl. Fig. 1 (b)) have dispersions $\hbar\omega_0 = \hbar\omega_0(k) = \Delta + \sqrt{\Delta^2 + (v_0 p)^2}$ and $\hbar\omega_0 = -\hbar\omega_0(k)$, and gapless modes (Suppl. Fig. 1 (c)) have dispersions $\hbar\omega_0 = \hbar\omega_0(k) = \Delta - \sqrt{\Delta^2 + (v_0 p)^2}$ and $\hbar\omega_0 = -\hbar\omega_0(k)$.

In the absence of the confinement ($g = 0$), the massless linear dispersion, $\omega_0 = \pm v_0 k$, is expected and there are 2 branches: one for a right moving photon and the other is for a left moving photon. The phase velocity is simply obtained $v_\mathrm{p} = c/n(\omega) = \omega_0(k)/k$, while the group velocity [3, 2] is calculated by the derivative $v_\mathrm{g} = (d\omega_0/dk)_{\omega_0}$. There is no difference in sign between v_p and v_g, but v_g is always smaller than v_p in the rest frame. For the complete linear dispersion at $g = 0$, these velocities are the same, $v_\mathrm{p} = v_\mathrm{g}$.

We consider a parity exchange operation in the momentum space, $\hat{\mathcal{P}}_k k = -k$, which converts the direction of the propagation. For $g = 0$, the branch of the dispersion is not changed (Suppl. Fig. 1 (a)), and thus we find $\hat{\mathcal{P}}_k \omega_0 = -\omega_0$, and $\hat{\mathcal{P}}_k v_p = v_p$. We also consider the parity operation to the chirality, χ_z, which is the projection of the spin expectation values $\mathbf{S} = \langle(\hat{\mathbf{S}})\rangle = (\langle \hat{S}_x\rangle, \langle \hat{S}_y\rangle, \langle \hat{S}_z\rangle)$ to the direction of the propagation [42, 49, 26]. The spin state changes the sign $\hat{\mathcal{P}}_k S_z = -S_z$, because of the change of the sign of the frequency, which changes the left circulation to the right circulation and *vice versa*. On the other hand, the direction of the propagation is preserved upon the parity operation within the branch (Suppl. Fig. 1 (a)), such that the chirality is the same sign with S_z as $\hat{\mathcal{P}}_k \chi_z = -\chi_z$.

In the GRIN fibre $g \neq 0$, the guided modes have an energy gap, Δ, (Suppl. Fig. 1 (b)), which can be regarded as a coupling between left and right moving modes, as we shall see in the next section. This is very similar to superconductivity, explained by the theory to form a Cooper pair between electrons with the opposite momentum [50, 51]. It also has a similar mathematical feature with the Tomonaga-Luttinger liquid for $1D$ electron systems [52, 53, 54]. Consequently, the branch exchange is expected upon the parity operation (Suppl. Fig. 1 (b)), $\hat{\mathcal{P}}_k k = -k$, such that the energy will not change the sign $\hat{\mathcal{P}}_k \omega_0 = \omega_0$, which is remarkable difference from a free photon. Therefore, the spin is pointing to the

same direction $\hat{\mathcal{P}}_k S_z = S_z$. On the other hand, the direction of the propagation is flipped $\hat{\mathcal{P}}_k v_p = -v_p$, as before, such that the chirality is also flipped $\hat{\mathcal{P}}_k \chi_z = -\chi_z$.

For the gapless mode (Suppl. Fig. 1(c)), we expect the radiative solution, as we discussed in the previous section. We also expect the same parity operations with the case for the gapped mode, because the spectra depends quadratic on $p = \hbar k$. The summary of the parity operation is given in Table 2.

If we consider both guided and radiative modes, we have 8 possible solutions for the Helmholtz equation of the GRIN fibre, among which 4 solutions are physical feasible (Table 3). There are 2 possibilities in the spectra: One is opening a gap, $\hbar\omega_{\rm gap} = \Delta + \sqrt{\Delta^2 + (v_0 p)^2}$, and the other is gapless, $\hbar\omega_{\rm gapless} = -\Delta + \sqrt{\Delta^2 + (v_0 p)^2}$. The angular frequency can be positive or negative (2 possibilities), and the final 2 possibilities are guided or radiative. The forms of the wavefunctions are summarised in Table 3.

If we define the time reversal operator as $\hat{\mathcal{T}} t = -t$, the existence of these solutions correspond to the time reversal symmetry of the system $\hat{\mathcal{T}} {\rm e}^{ikz - i\omega_{\rm gap} t} = {\rm e}^{ikz + i\omega_{\rm gap} t}$, and $\hat{\mathcal{T}} {\rm e}^{ikz - i\omega_{\rm gapless} t} = {\rm e}^{ikz + i\omega_{\rm gapless} t}$.

For photonic systems without gain or loss, the time reversal symmetry is guaranteed, and we always have 2 conjugate solutions like ${\rm e}^{ikz - i\omega t}$ and ${\rm e}^{-ikz + i\omega t}$. These solutions are obtained by the successive applications of parity-flip and time-reversal operations $\hat{\mathcal{P}}\hat{\mathcal{T}} {\rm e}^{ikz - i\omega t} = {\rm e}^{-ikz + i\omega t}$. The simultaneous existence of these solutions is required to guarantee the observable nature of electromagnetic fields, since these fields must be real [2, 1, 26]. While the behaviours of the plane waves against these operations are rather trivial, the impacts on spin and chirality are not trivial. We will focus on the quantum-mechanical origin of spin for a photon in the next section.

2.2. Polarisation state described by the 2D Dirac equation: The simplest example

Now, it is ready to discuss the polarisation state derived by the $2D$ Dirac equation. Here, we consider a free propagation limit of $\Delta = 0$. In this case, the Hamiltonians become

$$H_{\rm L} = \begin{pmatrix} \xi & 0 \\ 0 & -\xi \end{pmatrix} \quad (2)$$

$$H_{\rm R} = -\begin{pmatrix} \xi & 0 \\ 0 & -\xi \end{pmatrix}. \quad (3)$$

For the standard particle state along the time evolution of $t > 0$, the solutions are given by with $\hbar\omega = \xi > 0$, and we obtain

$$| \uparrow \rangle = |\mathrm{L}\rangle = \begin{pmatrix} 1 \\ 0 \end{pmatrix} \tag{4}$$

for H_{L} and

$$| \downarrow \rangle = |\mathrm{R}\rangle = \begin{pmatrix} 0 \\ 1 \end{pmatrix} \tag{5}$$

for H_{R}. We can construct the arbitrary polarisation state by the superposition state of these states, $|\mathrm{L}\rangle$ and $|\mathrm{R}\rangle$. The energy of $|\mathrm{L}\rangle$ and $|\mathrm{R}\rangle$ are the same, such that the superposition states of these states are also the same. For example, we obtain linearly polarised states along horizontal (H), vertical (V), diagonal (D), and anti-diagonal (A) directions as

$$| \mathrm{H} \rangle = \frac{1}{\sqrt{2}} \begin{pmatrix} 1 \\ 1 \end{pmatrix} \tag{6}$$

$$| \mathrm{V} \rangle = \frac{-i}{\sqrt{2}} \begin{pmatrix} 1 \\ -1 \end{pmatrix} \tag{7}$$

$$| \mathrm{D} \rangle = \frac{\mathrm{e}^{-\frac{\pi}{4}i}}{\sqrt{2}} \begin{pmatrix} 1 \\ i \end{pmatrix} \tag{8}$$

$$| \mathrm{A} \rangle = \frac{\mathrm{e}^{\frac{\pi}{4}i}}{\sqrt{2}} \begin{pmatrix} 1 \\ -i \end{pmatrix}, \tag{9}$$

respectively.

We can confirm that the superposition state is actually the steady state of the $2D$ Dirac equation without the time evolution. For example, for the horizontally polarised state, the wavefunction becomes

$$\psi_z(t) = \langle t|\mathrm{H}\rangle = \frac{\mathrm{e}^{ikz-i\omega t}}{\sqrt{2}} \begin{pmatrix} 1 \\ 1 \end{pmatrix}, \tag{10}$$

where $\hbar\omega = \xi$, and we confirmed it satisfies the $2D$ Dirac equation.

We have previously shown that the quantum-mechanical average of the spin operators correspond to the Stokes parameters [1]. For the horizontally polarised state, it becomes

$$\langle \mathbf{S} \rangle = \begin{pmatrix} \langle \sigma_{\mathbf{x}} \rangle \\ \langle \sigma_{\mathbf{y}} \rangle \\ \langle \sigma_{\mathbf{z}} \rangle \end{pmatrix} = \begin{pmatrix} \psi_z^*(t)\sigma_{\mathbf{x}}\psi_z(t) \\ \psi_z^*(t)\sigma_{\mathbf{y}}\psi_z(t) \\ \psi_z^*(t)\sigma_{\mathbf{z}}\psi_z(t) \end{pmatrix} = \begin{pmatrix} 1 \\ 0 \\ 0 \end{pmatrix}, \tag{11}$$

which is independent on t.

Similarly, the vertically polarised state becomes

$$\psi_z(t) = \langle t|\mathrm{V}\rangle = -i\frac{\mathrm{e}^{ikz-i\omega t}}{\sqrt{2}}\begin{pmatrix} 1 \\ -1 \end{pmatrix}, \tag{12}$$

which also satisfies the $2D$ Dirac equation. We also confirm that the time independent polarisation state

$$\langle \mathbf{S}\rangle = \begin{pmatrix} -1 \\ 0 \\ 0 \end{pmatrix}. \tag{13}$$

Here, we must emphasize the decoupling of the $2D$ Dirac equation into 2 equations

$$[i\hbar\partial_t - H_{\mathrm{L}}]\,\psi_z = 0 \tag{14}$$

$$[i\hbar\partial_t - H_{\mathrm{R}}]\,\psi_z = 0 \tag{15}$$

is not always true (Suppl. Fig. 2). In fact, the polarisation states $\langle t|\mathrm{H}\rangle$ and $\langle t|\mathrm{V}\rangle$ do not satisfy these equations at all, individually, while they satisfy the $2D$ Dirac equation (Suppl. Fig. 2 (a)) in the form of the successive applications of operators as a product, $[i\hbar\partial_t - H_{\mathrm{L}}]\,[i\hbar\partial_t - H_{\mathrm{R}}]$.

This could be confirmed by the example of a problem with the time evolution solely from H_{L}. In this case the 2 eigenvalues of $\hbar\omega = \xi$ and $-\hbar\omega = -\xi$ are assigned for $\langle t|\mathrm{L}\rangle$ and $\langle t|\mathrm{R}\rangle$, respectively. Then, the wavefunction at time of t is given by

$$\psi_z(t) = \langle t|\psi_z\rangle = \mathrm{e}^{-i\omega t}C_{\mathrm{L}}\langle 0|\mathrm{L}\rangle + \mathrm{e}^{+i\omega t}C_{\mathrm{R}}\langle 0|\mathrm{R}\rangle, \tag{16}$$

where C_{L} and C_{R} are complex coefficients determined by the initial condition ($t = 0$), which is given by

$$\psi_z(0) = C_{\mathrm{L}}\langle 0|\mathrm{L}\rangle + C_{\mathrm{R}}\langle 0|\mathrm{R}\rangle = \begin{pmatrix} C_{\mathrm{L}} \\ C_{\mathrm{R}} \end{pmatrix}. \tag{17}$$

Assuming the initial state is $\psi_z(0) = \langle t|\mathrm{H}\rangle$, we obtain

$$\psi_z(t) = \frac{\mathrm{e}^{-i\omega t}}{\sqrt{2}}\langle 0|\mathrm{L}\rangle + \frac{\mathrm{e}^{+i\omega t}}{\sqrt{2}}\langle 0|\mathrm{R}\rangle = \frac{1}{\sqrt{2}}\begin{pmatrix} \mathrm{e}^{-i\omega t} \\ \mathrm{e}^{+i\omega t} \end{pmatrix}, \tag{18}$$

which means that the state is not an steady state. In fact, the expectation values of the spin operators become

$$\langle \mathbf{S} \rangle = \begin{pmatrix} \cos(2\omega t) \\ \sin(2\omega t) \\ 0 \end{pmatrix}, \tag{19}$$

which describes the incoherent left circulation (Suppl. Fig. 2 (b)). Obviously, this is in contradiction with the fact that the horizontally polarised state is maintained in a free space. Similarly, if we use H_{R} instead of H_{L}, we found the opposite right circulation in the polarisation state (Suppl. Fig. 2 (c)) as

$$\langle \mathbf{S} \rangle = \begin{pmatrix} \cos(-2\omega_1 t) \\ \sin(-2\omega_1 t) \\ 0 \end{pmatrix}. \tag{20}$$

The problem arose, because of the incoherent superposition between states with the positive and negative energies, which would not be realised. The time evolution of these states will be opposite, so that we cannot consider the superposition states. As far as we consider the states with the proper time evolution ($t > 0$) and the superposition state of these states, we can decouple the product.

2.3. Rotation in spin state for photons: BCS-Anderson theory and Bogoliubov transformation

In the the previous sections, we have derived the fundamental $2D$ Dirac equation and the spin of photons for describing the polarisation state for a coherent ray of photons propagating in a GRIN fibre. In particular, the Hamiltonian we obtained has exactly the same structure of the BCS theory of superconductivity [50, 51]. At the early stage of the theory of superconductivity, Anderson also identified that the proposed variational state is essentially equivalent to a 2-level system, described by the superposition state between paired states and empty states, similar to the analogy to the spin density wave [55]. It is also identified by Bogoljubov[56] that the quasi-particle states are transferred to the different state with the energy gap upon the superconducting phase transition, and the unitary transformation is known as Bogoljubov transformation [56]. Mathematically, the highlight is simply described by the diagonalisation of 2×2 matrix. Our Hamiltonian also has the same structure, described by a 2-level system, because of the nature of SU(2) spin state of a photon [1]. Therefore, we employ the BCS-Anderson theory and Bogoljubov transformation for understanding the polarisation state of photons.

In the previous section, we used a chiral representation, for which left and right circularly polarised states are assigned to be $|\mathrm{L}\rangle = (1,0)$ and $|\mathrm{R}\rangle = (0,1)$ and the corresponding Stokes parameters of these states are $\mathbf{S} = (0,0,1)$ and $\mathbf{S} = (0,0,-1)$, respectively [1]. In the free space at $\Delta = 0$, the polarisation eigenstate for H_{L} with the energy of $\hbar\omega = \xi$ was located at $\mathbf{S} = (0,0,1)$ and the effective magnetic field was $\mathbf{h} = (0,0,\xi)$, pointing along S_3 (Suppl. Fig. 3). We consider what happens, if the confinement energy is finite, $\Delta \neq 0$. We use the SU(2) Lie algebra to obtain the spinor wavefunction [1].

We consider the proper time evolution by H_{L} with the positive energy state, which is the state, pointing along the direction of the effective magnetic field, $\mathbf{h} = (\Delta,0,\xi)$, on the Poincaré sphere (Suppl. Fig. 3). The rotation operator along S_2 is given by

$$\mathcal{D}_2(\theta_{\mathrm{L}}) = \mathcal{D}(\hat{\mathbf{y}},\theta_{\mathrm{L}}) = \exp\left(-\frac{i\sigma_y\theta_{\mathrm{L}}}{2}\right) = \begin{pmatrix} u_{\mathrm{L}} & -v_{\mathrm{L}} \\ v_{\mathrm{L}} & u_{\mathrm{L}} \end{pmatrix}, \tag{21}$$

where θ_{L} is the angle of the rotation, $\mathbf{1}$ is the 2×2 unit matrix in the spinor representation, $u_{\mathrm{L}} = \cos(\theta_{\mathrm{L}}/2)$, and $u_{\mathrm{R}} = \sin(\theta_{\mathrm{R}}/2)$. The normalisation condition,

$$|u_{\mathrm{L}}|^2 + |v_{\mathrm{L}}|^2 = 1, \tag{22}$$

is already satisfied.

In general for quantum mechanics, the matrix element of the Hamiltonian should not depend on the choice of the basis [11, 12]. Therefore, the unitary transformation, including a rotation, should preserve the matrix element. For example, if we consider a state given by $\langle z_i|$ and $|z_j\rangle$, the matrix element of the Hamiltonian is

$$\begin{aligned}\langle z_i|H|z_j\rangle &= \langle z_i|\mathcal{D}_y(\theta_{\mathrm{L}})\mathcal{D}_y^\dagger(\theta_{\mathrm{L}})H\mathcal{D}_y(\theta_{\mathrm{L}})\mathcal{D}_y^\dagger(\theta_{\mathrm{L}})|z_j\rangle & (23)\\ &= \langle z'_i|\mathcal{D}_y^\dagger(\theta_{\mathrm{L}})H\mathcal{D}_y(\theta_{\mathrm{L}})|z'_j\rangle & (24)\\ &= \langle z'_i|H'|z'_j\rangle. & (25)\end{aligned}$$

Therefore, the unitary transformation will rotate the basis state in the opposite way,

$$|z'\rangle = \mathcal{D}_y^\dagger(\theta_{\mathrm{L}})|z\rangle, \tag{26}$$

while the Hamiltonian is transferred as $\mathcal{D}_y^\dagger(\theta_{\mathrm{L}})H_{\mathrm{L}}\mathcal{D}_y(\theta_{\mathrm{L}})$. For our case, this becomes

$$\mathcal{D}_y^\dagger(\theta_{\mathrm{L}})H_{\mathrm{L}}\mathcal{D}_y(\theta_{\mathrm{L}}) = \begin{pmatrix} \xi\cos\theta_{\mathrm{L}} + \Delta\sin\theta_{\mathrm{L}} & -\xi\sin\theta_{\mathrm{L}} + \Delta\cos\theta_{\mathrm{L}} \\ -\xi\sin\theta_{\mathrm{L}} + \Delta\cos\theta_{\mathrm{L}} & -\xi\cos\theta_{\mathrm{L}} - \Delta\sin\theta_{\mathrm{L}} \end{pmatrix}. \tag{27}$$

By eliminating the off-diagonal component, we obtain the gap equation,

$$\tan\theta_{\mathrm{L}} = \frac{\Delta}{\xi}, \tag{28}$$

to diagonalise the Hamiltonian. The graphical representation of θ_{L} is shown in Suppl. Fig. 3. The gap equation gives

$$\cos\theta_{\mathrm{L}} = \frac{\xi}{\sqrt{\xi^2+\Delta^2}} \tag{29}$$

$$\sin\theta_{\mathrm{L}} = \frac{\Delta}{\sqrt{\xi^2+\Delta^2}}, \tag{30}$$

and consequently,

$$\xi\cos\theta_{\mathrm{L}} + \Delta\sin\theta_{\mathrm{L}} = \sqrt{\xi^2+\Delta^2}. \tag{31}$$

Thus, we diagonalised the Hamiltonian as

$$\mathcal{D}_y^{\dagger}(\theta_{\mathrm{L}})H_{\mathrm{L}}\mathcal{D}_y(\theta_{\mathrm{L}}) = \sqrt{\xi^2+\Delta^2}\begin{pmatrix} 1 & 0 \\ 0 & -1 \end{pmatrix} = \sqrt{\xi^2+\Delta^2}\sigma_z. \tag{32}$$

Alternatively, the 2×2 Hamiltonian could be simply diagonalised from the vanishing determinant as

$$\begin{vmatrix} \xi - E & \Delta \\ \Delta & -\xi - E \end{vmatrix} = E^2 - \xi^2 - \Delta^2 = 0, \tag{33}$$

which gives the famous quasi-particle dispersion of

$$E = \pm\sqrt{\xi^2+\Delta^2}. \tag{34}$$

It is also useful to note that we obtained the rotational parameters as

$$u_{\mathrm{L}}^2 = \cos^2\left(\frac{\theta_{\mathrm{L}}}{2}\right) = \frac{1}{2}(1+\cos\theta_{\mathrm{L}}) = \frac{1}{2}\left(1+\frac{\xi}{\sqrt{\xi^2+\Delta^2}}\right) \tag{35}$$

$$v_{\mathrm{L}}^2 = \sin^2\left(\frac{\theta_{\mathrm{L}}}{2}\right) = \frac{1}{2}(1-\cos\theta_{\mathrm{L}}) = \frac{1}{2}\left(1-\frac{\xi}{\sqrt{\xi^2+\Delta^2}}\right). \tag{36}$$

The spinor wavefunction for $E = \sqrt{\xi^2 + \Delta^2}$ is given by

$$u_\uparrow = \mathcal{D}_y(\theta_\mathrm{L}) \left(\begin{array}{c} 1 \\ 0 \end{array} \right) = \left(\begin{array}{c} u_\mathrm{L} \\ v_\mathrm{L} \end{array} \right) = \left(\begin{array}{c} \cos\left(\frac{\theta_\mathrm{L}}{2}\right) \\ \sin\left(\frac{\theta_\mathrm{L}}{2}\right) \end{array} \right), \quad (37)$$

for the proper time evolution, while the wavefunction for the time-reversal evolution is given by

$$u_\downarrow = \mathcal{D}_y(\theta_\mathrm{L}) \left(\begin{array}{c} 0 \\ 1 \end{array} \right) = \left(\begin{array}{c} -v_\mathrm{L} \\ u_\mathrm{L} \end{array} \right) = \left(\begin{array}{c} -\sin\left(\frac{\theta_\mathrm{L}}{2}\right) \\ \cos\left(\frac{\theta_\mathrm{L}}{2}\right) \end{array} \right), \quad (38)$$

for $E = -\sqrt{\xi^2 + \Delta^2}$.

The diagonalised solution is summarised in Table 4. We have assumed that the original basis state before the rotation ($\Delta = 0$) is assumed to be $|\mathrm{L}\rangle$ at $\mathbf{S} = (0, 0, 1)$. Using this original basis, we obtained the spinor wavefunction by the rotation in SU(2) Hilbert space. We can also re-define this rotated state as $|\mathrm{L}\rangle = (1, 0)$ for a new diagonal basis (Table 4) after the Bogoljubov transformation [56], described above for photons. We also obtained the conjugate solution for the time-reversal evolution.

In the strong confinement limit of $\xi \to 0$, the propagation along z is limited by the heavy effective mass of m^* and the dynamics of photons is dominated by the circular motion with orbital angular momentum and radial oscillation. Then, the energy is dominated by Δ, and the original $|\mathrm{L}\rangle$ would be rotated with the rotational angle of $\pi/2$ to be $|\mathrm{H}\rangle$ at $\mathbf{S} = (1, 0, 0)$ (Suppl. Fig. 3) by H_L.

Similarly, we consider the time evolution by $H_\mathrm{R} = -H_\mathrm{L}$. The solution could be easily obtained by recognising the mapping of

$$\Delta \rightarrow -\Delta \quad (39)$$

$$\xi \rightarrow -\xi, \quad (40)$$

from H_L to H_R. This will give the gap equation for θ_R as

$$\tan\theta_\mathrm{R} = \frac{\Delta}{\xi}, \quad (41)$$

which provides

$$\cos\theta_\mathrm{R} = -\frac{\xi}{\sqrt{\xi^2 + \Delta^2}} = -\cos\theta_\mathrm{L} \quad (42)$$

$$\sin\theta_\mathrm{R} = -\frac{\Delta}{\sqrt{\xi^2 + \Delta^2}} = -\sin\theta_\mathrm{L}. \quad (43)$$

Therefore, we obtained the relationship of

$$\theta_{\mathrm{R}} = \theta_{\mathrm{L}} + \pi. \tag{44}$$

The summary of the solutions for H_{R} is shown in Table 5. We obtained $|\mathrm{R}\rangle = (0, 1)$ for a standard time evolution. As a result, we obtained both $|\mathrm{L}\rangle$ and $|\mathrm{R}\rangle$ for the same energy of $E = \sqrt{\xi^2 + \Delta^2} > 0$. An arbitrary polarised state can be constructed by the superposition state of these orthogonal states [1]. Therefore, the Dirac equation can describe the time evolution of arbitrary polarised states for photons propagating in a GRIN fibre.

2.4. Freedom to assign polarisation state

So far, we have used a chiral representation for the basis states of $|\mathrm{L}\rangle$ and $|\mathrm{R}\rangle$ for describing the polarisation states of $(1, 0)$ and $(0, 1)$ [1]. We also call this basis as the LR-basis [1]. The Stokes parameters are given by

$$\mathbf{S} = S_0 \begin{pmatrix} 1 \\ \langle\sigma_x\rangle \\ \langle\sigma_y\rangle \\ \langle\sigma_z\rangle \end{pmatrix}, \tag{45}$$

where $S_0 = \hbar\mathcal{N}$ is the time-averaged total magnitude of the spin angular momentum for the number of photons of $\mathcal{N}$ in the LR-basis. However, the original Helmholtz equation for the wavefunction of photons do not have any particular preferential direction for the polarisation states. Consequently, we should preserve the rotational symmetry of the polarisation states. Therefore, the polarisation state should not be dependent on any particular choice of the basis states.

In Jones vector representation [8, 22, 2, 4, 5], we use $|\mathrm{H}\rangle = (1, 0)$ and $|\mathrm{V}\rangle = (0, 1)$ for the basis states, and we are calling as the HV-basis [1]. In this representation, the Stokes parameters are

$$\mathbf{S} = S_0 \begin{pmatrix} 1 \\ \langle\sigma_z\rangle \\ \langle\sigma_x\rangle \\ \langle\sigma_y\rangle \end{pmatrix}. \tag{46}$$

In our formulation, there is no difference at all, whether we should use LR or HV basis. We can also use the diagonal basis states, composed of $|\mathrm{D}\rangle = (1, 0)$ and $|\mathrm{A}\rangle = (0, 1)$. In the end, we obtain the degenerate energy states, which are mutually orthogonal. Arbitrary polarised states can be constructed by the superposition of these orthogonal basis states, such that the final state is not polarisation dependent.

2.5. Difference between SU(2) and SO(3)

Here, we briefly mention about the phase change upon 1-rotation on the Poincaré sphere [57, 58], which is coming from the difference between SU(2) for the wavefunction and SO(3) for the spin expectation value. The phase change is very important to consider the quantum-mechanical commutation relationship of spin operators for photons [1, 23, 26], and its impact on the interference [59, 60, 61].

In the gap equation, we only obtained $\tan\theta_{\rm L}$ or $\tan\theta_{\rm R}$ for $H_{\rm L}$ and $H_{\rm R}$, respectively. Therefore, in addition to the solutions of $\theta_{\rm L}$ and $\theta_{\rm R}$, we also have alternative solutions of $\theta_{\rm L}+\pi$ and $\theta_{\rm R}+\pi$. These solutions correspond to the change of $\xi \to -\xi$ and $\Delta \to -\Delta$, and therefore, they merely change the overall sign of the spinor wavefunction, $u_\sigma \to -u_\sigma$ for both polarisations of $\sigma = \uparrow$ and $\sigma = \downarrow$. In the diagonal basis, these just correspond to the changes of $|{\rm L}\rangle \to -|{\rm L}\rangle$ and $|{\rm R}\rangle \to -|{\rm R}\rangle$. This is similar to a Pancharatnam-Berry's phase [57, 58, 1].

For the polarisation state, described by the Stokes parameters of $\mathbf{S} = (S_1, S_2, S_3)$, there is no difference at all for the difference of the phase. Nevertheless, this phase is observable by interference experiments [59, 60, 61, 1]. In the BCS theory of superconductivity, this degree of freedom is hidden in a superconducting order parameter as a phase degree of freedom [62, 55, 63, 64, 51]. Our formulation of the Dirac equation for photons has the same mathematical structure, and we will show the importance of the phase degree of freedom in the order parameter of Δ, in the next section.

2.6. Poincaré sphere

Here, we discuss the importance of the phase in the energy gap, and its impact on the polarisation states. We discuss the principle of rotational symmetry in polarisation states and the role of the phase of the energy gap, which corresponds to the azimuthal angle. First, we go back to the Klein-Gordon equation and discuss the freedom to assign Pauli spin matrices. We consider another choice of

$$\alpha_x = \sigma_y = \begin{pmatrix} 0 & -i \\ i & 0 \end{pmatrix} \tag{47}$$

$$\alpha_z = \sigma_z = \begin{pmatrix} 1 & 0 \\ 0 & -1 \end{pmatrix}. \tag{48}$$

In this choice of the gauge, we obtain the Dirac equation as

$$\left(i\hbar\partial_t - \xi\sigma_z - \Delta\sigma_y\right)\left(i\hbar\partial_t + \xi\sigma_z + \Delta\sigma_y\right)\psi_z = 0, \tag{49}$$

using the same parameters of $\xi = v_0 p$ and $\Delta = m^* v_0^2$. The only difference from the previous choice (Suppl. Fig. 4) is the direction of the effective magnetic field as $\mathbf{h} = (0, \Delta, \xi)$.

In this case, for the solution of the de-coupled Dirac equation of H_{L}, the eigenstate pointing $\mathbf{h}$ is obtained by the rotation along S_1 in the clock-wise direction, seen from the top of the S_1 axis with θ_{L}, which is given by the SU(2) rotation operator,

$$\mathcal{D}_1(-\theta_{\mathrm{L}}) = \mathcal{D}(\hat{\mathbf{x}}, -\theta_{\mathrm{L}}) = \exp\left(+\frac{i\sigma_x \theta_{\mathrm{L}}}{2}\right) = \begin{pmatrix} u_{\mathrm{L}} & iv_{\mathrm{L}} \\ iv_{\mathrm{L}} & u_{\mathrm{L}} \end{pmatrix}. \tag{50}$$

The rotated Hamiltonian becomes

$$\mathcal{D}_x^{\dagger}(-\theta_{\mathrm{L}}) H_{\mathrm{L}} \mathcal{D}_x(-\theta_{\mathrm{L}}) = \begin{pmatrix} \xi\cos\theta_{\mathrm{L}} + \Delta\sin\theta_{\mathrm{L}} & i\xi\sin\theta_{\mathrm{L}} - i\Delta\cos\theta_{\mathrm{L}} \\ -i\xi\sin\theta_{\mathrm{L}} + i\Delta\cos\theta_{\mathrm{L}} & -\xi\cos\theta_{\mathrm{L}} - \Delta\sin\theta_{\mathrm{L}} \end{pmatrix}. \tag{51}$$

By eliminating the off-diagonal component, we obtain the same gap equation of

$$\tan\theta_{\mathrm{L}} = \frac{\Delta}{\xi} \tag{52}$$

as in the previous choice, such that the angle θ_{L} is not changed. Consequently, we obtain the same eigenvalues of $E = \pm\sqrt{\xi^2 + \Delta^2}$. However, we obtain slightly different eigenfunctions

$$u_{\uparrow} = \mathcal{D}_x(-\theta_{\mathrm{L}}) \begin{pmatrix} 1 \\ 0 \end{pmatrix} = \begin{pmatrix} u_{\mathrm{L}} \\ iv_{\mathrm{L}} \end{pmatrix} = \begin{pmatrix} \cos\left(\frac{\theta_{\mathrm{L}}}{2}\right) \\ i\sin\left(\frac{\theta_{\mathrm{L}}}{2}\right) \end{pmatrix} \tag{53}$$

$$u_{\downarrow} = \mathcal{D}_x(-\theta_{\mathrm{L}}) \begin{pmatrix} 0 \\ 1 \end{pmatrix} = \begin{pmatrix} iv_{\mathrm{L}} \\ u_{\mathrm{L}} \end{pmatrix} = \begin{pmatrix} i\sin\left(\frac{\theta_{\mathrm{L}}}{2}\right) \\ \cos\left(\frac{\theta_{\mathrm{L}}}{2}\right) \end{pmatrix}. \tag{54}$$

These states are orthogonal each other, as confirmed by the inner product

$$u_{\uparrow}^* u_{\downarrow} = \begin{pmatrix} \cos\left(\frac{\theta_{\mathrm{L}}}{2}\right) & -i\sin\left(\frac{\theta_{\mathrm{L}}}{2}\right) \end{pmatrix} \begin{pmatrix} i\sin\left(\frac{\theta_{\mathrm{L}}}{2}\right) \\ \cos\left(\frac{\theta_{\mathrm{L}}}{2}\right) \end{pmatrix} = 0. \tag{55}$$

For the standard time evolution of the energy of $E = \sqrt{\xi^2 + \Delta^2}$, we obtained

$$|\mathrm{L}\rangle = \begin{pmatrix} \cos\left(\frac{\theta_{\mathrm{L}}}{2}\right) \\ i\sin\left(\frac{\theta_{\mathrm{L}}}{2}\right) \end{pmatrix} \tag{56}$$

in the original chiral basis, if the magnetic field is applied to $\mathbf{h} = (0, \Delta, \xi)$. This is in contrast to the previous solution of

$$|\mathrm{L}\rangle = \begin{pmatrix} \cos\left(\frac{\theta_\mathrm{L}}{2}\right) \\ \sin\left(\frac{\theta_\mathrm{L}}{2}\right) \end{pmatrix} \tag{57}$$

for the field of $\mathbf{h} = (\Delta, 0, \xi)$.

The difference of the phase of i, could be understood by the rotation in the $S_1 - S_2$ plane, which is enabled by a rotator operator [1],

$$\mathcal{D}_3\left(\frac{\pi}{2}\right) = \exp\left(+\frac{i\sigma_z\pi}{4}\right) = \begin{pmatrix} \mathrm{e}^{-i\frac{\pi}{4}} & 0 \\ 0 & \mathrm{e}^{+i\frac{\pi}{4}} \end{pmatrix} = \mathrm{e}^{-i\frac{\pi}{4}} \begin{pmatrix} 1 & 0 \\ 0 & i \end{pmatrix}. \tag{58}$$

In fact, by applying this rotator, we confirm

$$\mathcal{D}_3\left(\frac{\pi}{2}\right)\begin{pmatrix} \cos\left(\frac{\theta_\mathrm{L}}{2}\right) \\ \sin\left(\frac{\theta_\mathrm{L}}{2}\right) \end{pmatrix} = \mathrm{e}^{-\frac{\pi}{4}i} \begin{pmatrix} \cos\left(\frac{\theta_\mathrm{L}}{2}\right) \\ i\sin\left(\frac{\theta_\mathrm{L}}{2}\right) \end{pmatrix}. \tag{59}$$

Therefore, the choice of the Pauli spin matrices are just coming from the choice of the polarisation axis in the $S_1 - S_2$ plane.

In order to confirm this view, we consider a more elaborate choice of the Pauli spin matrices for the derivation of the Dirac equation as

$$\alpha_z = \sigma_z = \begin{pmatrix} 1 & 0 \\ 0 & -1 \end{pmatrix} \tag{60}$$

$$\alpha_x = \sigma_x \cos\phi + \sigma_y \sin\phi = \begin{pmatrix} 0 & \mathrm{e}^{-i\phi} \\ \mathrm{e}^{i\phi} & 0 \end{pmatrix}, \tag{61}$$

where ϕ is the phase, describing the azimuthal angle on the Poincaré sphere (Suppl. Fig. 5). In addition to the trivial identity, $\sigma_z^2 = \mathbf{1}$, these choices in fact satisfy the splitting conditions,

$$\alpha_x^2 = \sigma_x \cos^2\phi + \sigma_y \sin^2\phi \cos\phi \sin\phi \left(\sigma_x\sigma_y + \sigma_y\sigma_x\right) = \mathbf{1} \tag{62}$$

$$\alpha_x\sigma_z = \sigma_x\sigma_z \cos\phi\sigma_y\sigma_z \sin\phi = -\sigma_z\left(\sigma_x \cos\phi\sigma_y \sin\phi\right) = -\sigma_z\alpha_x. \tag{63}$$

In this gauge, the $2D$ Dirac equation becomes

$$\left[i\hbar\partial_t - \xi\sigma_z - \Delta\left(\cos\phi\sigma_x + \sin\phi\sigma_y\right)\right]\left[i\hbar\partial_t + \xi\sigma_z + \Delta\left(\cos\phi\sigma_x + \sin\phi\sigma_y\right)\right]\psi_z \tag{64}$$

Thus, the magnetic field is $\mathbf{h} = (\Delta \cos\phi, \Delta \sin\phi, \xi)$. The Hamiltonian for the left state is

$$H_{\mathrm{L}} = \mathbf{h} \cdot \sigma = \begin{pmatrix} \xi & \Delta \mathrm{e}^{-i\phi} \\ \Delta \mathrm{e}^{i\phi} & -\xi \end{pmatrix}, \tag{65}$$

which is in fact Hermite

$$H_{\mathrm{L}}^{\dagger} = H_{\mathrm{L}} = H. \tag{66}$$

Similarly, the Hamiltonian for the right state is

$$H_{\mathrm{R}} = -H_{\mathrm{L}} = -\mathbf{h} \cdot \sigma = -\begin{pmatrix} \xi & \Delta \mathrm{e}^{-i\phi} \\ \Delta \mathrm{e}^{i\phi} & -\xi \end{pmatrix}. \tag{67}$$

We can now identify that the introduced angle of ϕ is actually the phase of the order parameter, $\Delta \mathrm{e}^{i\phi}$.

In order to eliminate the impact of the phase, we can rotate the polarisation state on the Poincaré sphere (Suppl. Fig. 5). To obtain the left circularly polarised state for $\Delta \mathrm{e}^{i\phi} \neq 0$ for H_{L}, first we rotate along the S_3 axis by

$$\mathcal{D}_3(\phi) \quad = \exp\left(-\frac{i\sigma_z \phi}{2}\right) = \begin{pmatrix} \mathrm{e}^{-i\frac{\phi}{2}} & 0 \\ 0 & \mathrm{e}^{+i\frac{\phi}{2}} \end{pmatrix}. \tag{68}$$

The rotated Hamiltonian would be

$$\mathcal{D}_z^{\dagger}(\phi) H_{\mathrm{L}} \mathcal{D}_z(\phi) = \begin{pmatrix} \xi & \Delta \\ \Delta & -\xi \end{pmatrix} = \xi \sigma_z + \Delta \sigma_x. \tag{69}$$

Thus, the impact of the complex nature of the order parameter is successfully eliminated. Subsequently, we rotate along the S_2 axis as

$$\mathcal{D}_y^{\dagger}(\theta_{\mathrm{L}}) \mathcal{D}_z^{\dagger}(\phi) H_{\mathrm{L}} \mathcal{D}_z(\phi) \mathcal{D}_y(\theta_{\mathrm{L}}) = \sqrt{\xi^2 + \Delta^2} \sigma_z \tag{70}$$

to obtain the diagonalised Hamiltonian. The corresponding eigenstate for the positive energy becomes

$$u_{\uparrow} \quad = \quad \mathcal{D}_z(\phi) \mathcal{D}_y(\theta_{\mathrm{L}}) \begin{pmatrix} 1 \\ 0 \end{pmatrix} = \begin{pmatrix} \mathrm{e}^{-i\frac{\phi}{2}} \cos\left(\frac{\theta_{\mathrm{L}}}{2}\right) \\ \mathrm{e}^{+i\frac{\phi}{2}} \sin\left(\frac{\theta_{\mathrm{L}}}{2}\right) \end{pmatrix}, \tag{71}$$

which is the state for the rotated $|\mathrm{L}\rangle$. The eigenstate for the negative energy becomes

$$u_\downarrow = \mathcal{D}_z(\phi)\mathcal{D}_y(\theta_\mathrm{L})\begin{pmatrix} 0 \\ 1 \end{pmatrix} = \begin{pmatrix} -\mathrm{e}^{-i\frac{\phi}{2}}\sin\left(\frac{\theta_\mathrm{L}}{2}\right) \\ \mathrm{e}^{+i\frac{\phi}{2}}\cos\left(\frac{\theta_\mathrm{L}}{2}\right) \end{pmatrix}, \tag{72}$$

which also corresponds to $|\mathrm{R}\rangle$ for the standard time evolution, described by H_R. These states are orthogonal each other

$$u_\uparrow^* u_\downarrow = \begin{pmatrix} \mathrm{e}^{i\frac{\phi}{2}}\cos\left(\frac{\theta_\mathrm{L}}{2}\right) & \mathrm{e}^{-i\frac{\phi}{2}}\sin\left(\frac{\theta_\mathrm{L}}{2}\right) \end{pmatrix}\begin{pmatrix} -\mathrm{e}^{-i\frac{\phi}{2}}\sin\left(\frac{\theta_\mathrm{L}}{2}\right) \\ \mathrm{e}^{+i\frac{\phi}{2}}\cos\left(\frac{\theta_\mathrm{L}}{2}\right) \end{pmatrix} = 0. \tag{73}$$

The obtained polarisation state is exactly the same form for the Bloch state in the 2-level system [1]. In fact, the polarisation state is described by quantum mechanical 2-level system with the SU(2) symmetry [21, 22, 8, 2, 11, 12, 1]. By making the superposition state of $|\mathrm{L}\rangle$ and $|\mathrm{R}\rangle$, we can construct any polarisation state.

Acknowledgements

This work was supported by JSPS KAKENHI Grant Number JP 18K19958. The author would like to express sincere thanks to Prof I. Tomita for continuous discussions and encouragements.

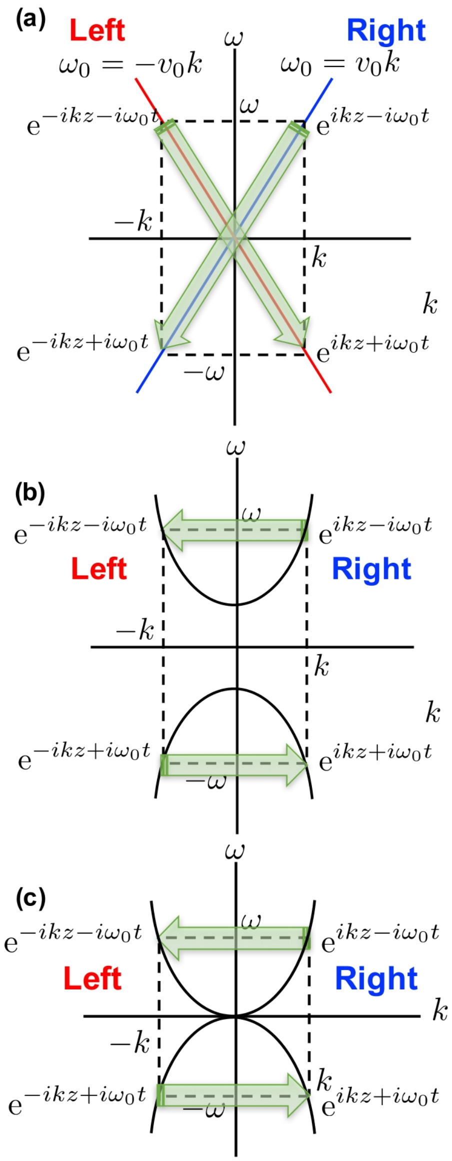

Supplementary Figure 1: Schematic dispersion relationships for a graded-index (GRIN) fibre. (a) In the free confinement limit ($g = 0$), the dispersions are just linear. 2 branches exist: one for a right propagating mode and the other is for a left propagation mode. There is no branch exchange upon the parity operation ($-k \leftrightarrow k$) in the momentum space. The original right propagating state is still describing for the right mode upon the parity operation, and *vice versa*. (b) In the GRIN fibre ($g \neq 0$), the guided mode has be opening up an energy gap and the spectra are massive. The parity operation makes the branch exchange from the right to the left and *vice versa*, while keeping the sign of the energy unchanged. The existence of the complex conjugate solution guarantees the observable nature of electromagnetic fields. (c) For the radiative modes from the GRIN fibre ($g \neq 0$), the energy spectra are gapless. Still, the branch exchange is expected upon the parity operation.

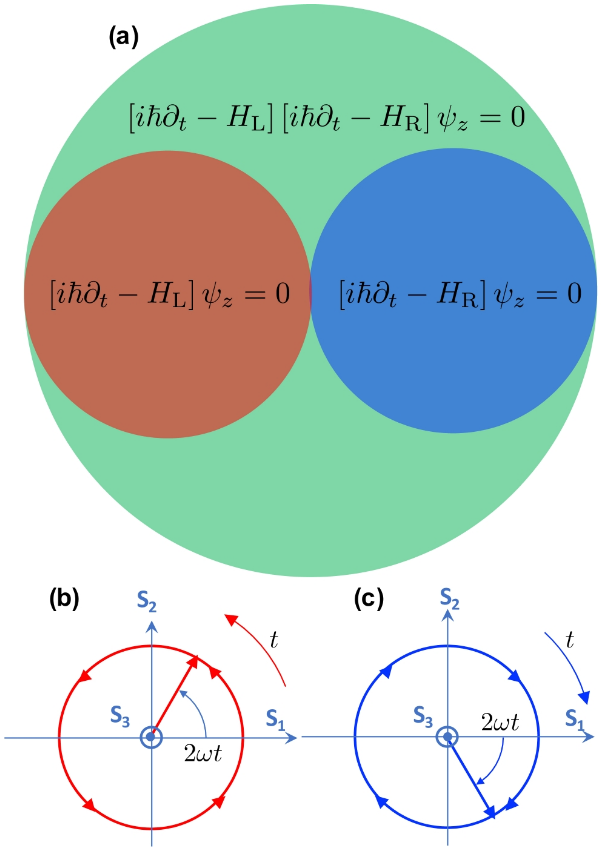

Supplementary Figure 2: Dirac equation for photons and de-coupling of the equation. (a) Schematic image of the Hilbert space spanned by the solution of the equations. If the de-coupled equation is satisfied, the Dirac equation is automatically satisfied. However, there exists solutions of the Dirac equation, without satisfying the de-coupled ones. For example, in the chiral representation, where $|\mathrm{L}\rangle = (1, 0)$ and $|\mathrm{R}\rangle = (0, 1)$ are eigenstates of H_L and H_R, respectively, these states satisfy the decoupled equations. However, the superposition state of $|\mathrm{H}\rangle \propto (1, 1)$ and $|\mathrm{V}\rangle \propto (1, -1)$ do not satisfy these de-coupled equations, but they satisfy the original Dirac equation. Therefore, the de-coupling is not always justified. (b) (c) Examples of incoherent states, which will not be realised. (b) The time evolution, expected solely from H_L. If we make a superposition state of among states with positive energy $\hbar\omega > 0$ and negative energy $-\hbar\omega < 0$, the polarisation state becomes rapidly rotating at 2ω towards the left circulation over time, seen from the top of the S_3 axis. (c) The rapid rotation of polarisation state, circulating to the right solely from H_R.

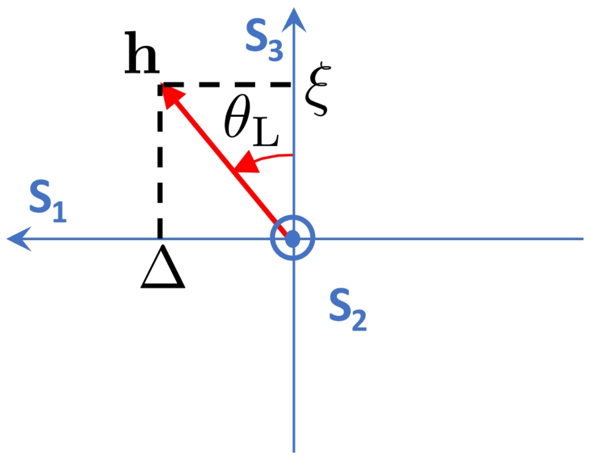

Supplementary Figure 3: Poincaré sphere, described by Stokes parameters, $\mathbf{S} = (S_1, S_2, S_3)$. The effective magnetic field $\mathbf{h} = (\Delta, 0, \xi)$ is applied to change the polarisation state. The original state of $|\mathrm{L}\rangle$ at $\Delta = 0$ is located at $(0, 0, 1)$ The eigenvalue for the proper time evolution is the state, pointing along $\mathbf{h}$, which is rotated along S_2 with the amount of θ_{L}.

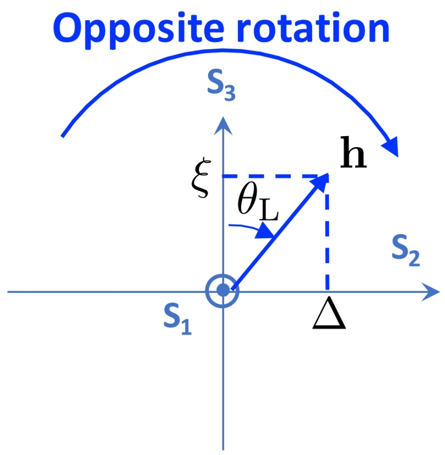

Supplementary Figure 4: Another choice of the gauge for the polarisation rotation on the Poincaré sphere. The effective magnetic field $\mathbf{h} = (0, \Delta, \xi)$ is applied to change the polarisation state. The rotation is along S_1 with the amount of $-\theta_{\mathrm{L}}$, which is the opposite direction with the previous choice.

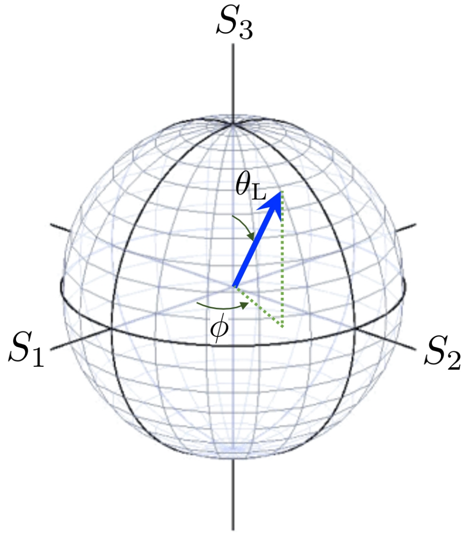

Supplementary Figure 5: Stokes parameters on the Poincaré sphere. The effective magnetic field, $\mathbf{h} = (\Delta \cos\phi, \Delta \sin\phi, \xi)$, is pointing to the direction of the polarisation state. The phase (ϕ) of the order parameter, $\Delta e^{i\phi} \neq 0$, is the azimuthal angle of the polarisation state. The polar angle (θ_{L}) is determined by the gap equation of $\tan(\theta_{\mathrm{L}}) = \Delta/\xi$, which is determined by the ratio of the energy for the confinement energy (orbital and radial contributions) and the kinetic energy along the direction of the propagation.

Table 1: Dispersion relationships for a graded-index (GRIN) fibre. The guided mode has a gapful dispersion for a standard time evolution, while the leaky mode is gapless. The reverse time is only meaningful theoretically, or it is valid for describing the light propagation to the opposite direction, $k < 0$.

	Standard time	Reverse time
ψ_z^+ (Guided)	$E_0^{++} > 0$ (Gapful)	$E_0^{+-} < 0$ (Gapless)
ψ_z^- (Leaky)	$E_0^{-+} > 0$ (Gapless)	$E_0^{--} < 0$ (Gapful)

Table 2: Parity operation for modes in a free space and a graded-index (GRIN) fibre.

	Free space	GRIN fibre
Momentum	$\hat{\mathcal{P}}_k k = -k$	$\hat{\mathcal{P}}_k k = -k$
Energy	$\hat{\mathcal{P}}_k \omega_0 = -\omega_0$	$\hat{\mathcal{P}}_k \omega_0 = \omega_0$
Velocity	$\hat{\mathcal{P}}_k v_p = v_p$	$\hat{\mathcal{P}}_k v_p = -v_p$
Chirality	$\hat{\mathcal{P}}_k \chi_z = -\chi_z$	$\hat{\mathcal{P}}_k \chi_z = -\chi_z$
Spin	$\hat{\mathcal{P}}_k S_z = -S_z$	$\hat{\mathcal{P}}_k S_z = S_z$

Table 3: Possible wavefunctions in a graded-index (GRIN) fibre. The major dependences of $\psi(r)\psi(z)$ on r, z, and t are shown. Physically unrealistic solutions are shown as n/a.

Dispersion	Guided	Radiative
$\omega_0 = \omega_{\mathrm{gap}} > 0$	$\mathrm{e}^{ikz - i\omega_{\mathrm{gap}} t} \mathrm{e}^{-\frac{r^2}{w_0^2}}$	n/a
$\omega_0 = \omega_{\mathrm{gapless}} > 0$	n/a	$\mathrm{e}^{ikz - i\omega_{\mathrm{gapless}} t} \mathrm{e}^{+\frac{r^2}{w_0^2}}$
$\omega_0 = -\omega_{\mathrm{gap}} < 0$	n/a	$\mathrm{e}^{ikz + i\omega_{\mathrm{gapless}} t} \mathrm{e}^{+\frac{r^2}{w_0^2}}$
$\omega_0 = -\omega_{\mathrm{gap}} < 0$	$\mathrm{e}^{ikz + i\omega_{\mathrm{gap}} t} \mathrm{e}^{-\frac{r^2}{w_0^2}}$	n/a

Table 4: Summary of the solution obtained from the decoupled Dirac equation for photons with time evolution by H_{L}. The standard time evolution is described by the eigenstate of the positive energy, while the conjugate solution with the negative energy is obtained for the time-reversal symmetry. The original spinor wavefunction is based on the basis with the left-circular polarised state of $|\mathrm{L}\rangle$ with $\mathbf{S} = (0, 0, 1)$ for $\Delta = 0$, and it was rotated on the Poincaré sphere for $\Delta \neq 0$. The diagonal basis is the basis to re-set the axis along the direction of the effective magnetic field.

Energy	Diagonal basis	Original basis
$\sqrt{\xi^2 + \Delta^2}$	$\lvert\mathrm{L}\rangle = \begin{pmatrix} 1 \\ 0 \end{pmatrix}$	$\begin{pmatrix} \cos\left(\frac{\theta_{\mathrm{L}}}{2}\right) \\ \sin\left(\frac{\theta_{\mathrm{L}}}{2}\right) \end{pmatrix}$
$-\sqrt{\xi^2 + \Delta^2}$	$\begin{pmatrix} 0 \\ 1 \end{pmatrix}$	$\begin{pmatrix} -\sin\left(\frac{\theta_{\mathrm{L}}}{2}\right) \\ \cos\left(\frac{\theta_{\mathrm{L}}}{2}\right) \end{pmatrix}$

Table 5: Summary of the solution obtained from the decoupled Dirac equation for photons with time evolution by H_{R}. There is a relationship between left and right rotation angles as $\theta_{\mathrm{R}} = \theta_{\mathrm{L}} + \pi$.

Energy	Diagonal basis	Original basis
$\sqrt{\xi^2 + \Delta^2}$	$\lvert\mathrm{R}\rangle = \begin{pmatrix} 0 \\ 1 \end{pmatrix}$	$\begin{pmatrix} \cos\left(\frac{\theta_{\mathrm{R}}}{2}\right) \\ \sin\left(\frac{\theta_{\mathrm{R}}}{2}\right) \end{pmatrix} = \begin{pmatrix} -\sin\left(\frac{\theta_{\mathrm{L}}}{2}\right) \\ \cos\left(\frac{\theta_{\mathrm{L}}}{2}\right) \end{pmatrix}$
$-\sqrt{\xi^2 + \Delta^2}$	$-\begin{pmatrix} 1 \\ 0 \end{pmatrix}$	$\begin{pmatrix} -\sin\left(\frac{\theta_{\mathrm{R}}}{2}\right) \\ \cos\left(\frac{\theta_{\mathrm{R}}}{2}\right) \end{pmatrix} = -\begin{pmatrix} \cos\left(\frac{\theta_{\mathrm{L}}}{2}\right) \\ \sin\left(\frac{\theta_{\mathrm{L}}}{2}\right) \end{pmatrix}$

References

[1] S. Saito, Quantum field theory for coherent photons: isomorphism between stokes parameters and spin expectation values, Front. Phys. 11 (2024) 1225334. doi:10.3389/fphy.2023.1225334.

[2] Y. Yariv, P. Yeh, Photonics: optical electronics in modern communications, Oxford University Press, Oxford, 1997.

[3] J. D. Jackson, Classical Electrodynamics, John Wiley & Sons, New York, 1999.

[4] D. H. Goldstein, Polarized Light, CRC Press, London, 2011. doi:10.1201/b10436.

[5] J. J. Gil, R. Ossikovski, Polarized Light and the Mueller Matrix Approach, CRC Press, London, 2016. doi:10.1201/b19711.

[6] F. L. Pedrotti, L. M. Pedrotti, L. S. Pedrotti, Introduction to Optics, Pearson Education, New York, 2007.

[7] E. Hecht, Optics, Pearson Education, Essex, 2017.

[8] R. C. Jones, A new calculus for the treatment of optical systems i. description and discussion of the calculus, J. Opt. Soc. Am. 31 (1941) 488–493. doi:10.1364/JOSA.31.000488.

[9] W. T. Payne, Elementary spinor theory, Am. J. Phys. 20 (1952) 253–262. doi:10.1119/1.1933190.

[10] M. Born, E. Wolf, Principles of Optics, Cambridge University Press, Cambridge, 1999. doi:10.1017/9781108769914.

[11] G. Baym, Lectures on Quantum Mechanics, Westview Press, New York, 1969.

[12] J. J. Sakurai, J. J. Napolitano, Modern Quantum Mechanics, Pearson, Edinburgh, 2014.

[13] E. Collett, Stokes parameters for quantum systems, Am. J. Phys. 38 (1970) 563–574. doi:10.1119/1.1976407.

[14] A. Luis, Degree of polarization in quantum optics, Phys. Rev. A 66 (2002) 013806. doi:10.1103/PhysRevA.66.013806.

[15] A. Luis, Polarization distributions and degree of polarization for quantum gaussian light fields, Opt. Comm. 273 (2007) 173–181. doi:10.1016/j.optcom.2007.01.016.

[16] G. Björk, J. Söderholm, L. L. Sánchez-Soto, A. B. Klimov, I. Ghiu, P. Marian, T. A. Marian, Quantum degrees of polarization, Opt. Comm. 283 (2010) 4440–4447. doi:10.1016/j.optcom.2010.04.088.

[17] G. F. T. d. Castillo, I. R. García, The Jones vector as a spinor and its representation on the Poincaré sphere, Rev. Mex. Fis. 57 (2011) 406–413. doi:https://rmf.smf.mx/ojs/index.php/rmf/article/view/3856.

[18] M. Sotto, I. Tomita, K. Debnath, S. Saito, Polarization rotation and mode splitting in photonic crystal line-defect waveguides, Front. Phys. 6 (2018) 85. doi:10.3389/fphy.2018.00085.

[19] M. Sotto, K. Debnath, A. Z. Khokhar, I. Tomita, D. Thomson, S. Saito, Anomalous zero-group-velocity photonic bonding states with local chirality, J. Opt. Soc. Am. B 35 (2018) 2356–2363. doi:10.1364/JOSAB.35.002356.

[20] M. Sotto, K. Debnath, I. Tomita, S. Saito, Spin-orbit coupling of light in photonic crystal waveguides, Phys. Rev. A 99 (2019) 053845. doi:10.1103/PhysRevA.99.053845.

[21] G. G. Stokes, On the composition and resolution of streams of polarized light from different sources, Trans. Cambridge Phil. Soc. 9 (1851) 399–416. doi:10.1017/CBO9780511702266.010.

[22] J. H. Poincaré, Théorie mathématique de la lumière, Tome 2, G. Carré, Paris, 1892. doi:https://gallica.bnf.fr/ark:/12148/bpt6k5462651m.

[23] S. Saito, Quantum commutation relationship for photonic orbital angular momentum, Front. Phys. 11 (2023) 1225346. doi:10.3389/fphy.2023.1225346.

[24] S. Saito, Nested SU(2) symmetry of photonic orbital angular momentum, Front. Phys. 11 (2023) 1289062. doi:10.3389/fphy.2023.1289062.

[25] L. Allen, M. W. Beijersbergen, R. J. C. Spreeuw, J. P. Woerdman, Orbital angular momentum of light and the transformation of Laguerre-Gaussian laser modes, Phys. Rev. A 45 (1992) 8185–8189. doi:10.1103/PhysRevA.45.8185.

[26] S. Saito, Spin and orbital angular momentum of coherent photons in a waveguide, Front. Phys. 11 (2023) 1225360. doi:10.3389/fphy.2023.1225360.

[27] A. M. Yao, M. J. Padgett, Orbital angular momentum: origins behavior and applications, Adv. Opt. Photon. 3 (2011) 161–204. doi:10.1364/AOP.3.000161.

[28] S. J. v. Enk, G. Nienhuis, Commutation rules and eigenvalues of spin and orbital angular momentum of radiation fields, J. Mod. Opt. 41 (1994) 963–977. doi:10.1080/09500349414550911.

[29] E. Leader, C. Lorcé, The angular momentum controversy: What's it all about and does it matter?, Phys. Rep. 541 (2014) 163–248. doi:10.1016/j.physrep.2014.02.010.

[30] S. M. Barnett, L. Allen, R. P. Cameron, C. R. Gilson, M. J. Padgett, F. C. Speirits, A. M. Yao, On the natures of the spin and orbital parts of optical angular momentum, J. Opt. 18 (2016) 064004. doi:10.1088/2040-8978/18/6/064004.

[31] G. Grynberg, A. Aspect, C. Fabre, Introduction to Quantum Optics: From the Semi-classical Approach to Quantized Light, Cambridge University Press, Cambridge, 2010.

[32] K. Y. Bliokh, F. J. Rodríguez-Fortuño, F. Nori, A. V. Zayats, Spin-orbit interactions of light, Nat. Photon. 9 (2015) 796–808. doi:10.1038/NPHOTON.2015.201.

[33] X. Ji, Comment on "Spin and orbital angular momentum in gauge theories: Nucleon spin structure and multipole radiation revisited", Phys. Rev. Lett. 104 (2010) 039101. doi:10.1103/PhysRevLett.104.039101.

[34] X. S. Chen, X. F. Lü, W. M. Sun, F. Wang, T. Goldman, Spin and orbital angular momentum in gauge theories: Nucleon spin structure

and multipole radiation revisited, Phys. Rev. Lett. 100 (2008) 232002. doi:10.1103/PhysRevLett.100.232002.

[35] L. P. Yang, F. Khosravi, Z. Jacob, Quantum field theory for spin operator of the photon, Phys. Rev. Research 4 (2022) 023165. doi:10.1103/PhysRevResearch.4.023165.

[36] E. S. Abers, Quantum Mechanics, Pearson, Los Angels, 2003.

[37] S. Kawakami, J. Nishizawa, An optical waveguide with the optimum distribution of the refractive index with reference to waveform distortion, IEEE Trans. Microw. Theory Techn. 16 (1968) 814–818. doi:10.1109/TMTT.1968.1126797.

[38] M. Plank, On the theory of the energy distribution law of the normal spectrum, Verhandl. Dtsch. Phys. Ges. 2 (1900) 237–245. doi:10.1016/B978-0-08-012102-4.50013-9.

[39] A. Einstein, Concerning an heuristic point of view toward the emission and transformation of light, Ann. Phys. 17 (1905) 132. doi:https://einsteinpapers.press.princeton.edu/papers.

[40] N. Bohr, The spectra of helium and hydrogen, Nature 92 (1913) 231–232. doi:10.1038/092231d0.

[41] P. A. M. Dirac, The Principle of Quantum Mechanics, Oxford University Press, Oxford, 1930.

[42] J. J. Sakurai, Advanced Quantum Mechanics, Addison-Wesley Publishing Company, New York, 1967.

[43] M. J. Padgett, J. Courtial, Poincaré-sphere equivalent for light beams containing orbital angular momentum, Opt. Lett. 24 (1999) 430–432. doi:10.1364/OL.24.000430.

[44] A. Holleczek, A. Aiello, C. Gabriel, C. Marquardt, G. Leuchs, Classical and quantum properties of cylindrically polarized states of light, Opt. Exp. 19 (2011) 9714–9736. doi:10.1364/OE.19.009714.

[45] G. Milione, H. I. Sztul, D. A. Nolan, R. R. Alfano, Higher-order poincaré sphere, stokes parameters, and the angular momentum of light, Phys. Rev. Lett. 107 (2011) 053601. doi:10.1103/PhysRevLett.107.053601.

[46] Z. Liu, Y. Liu, Y. Ke, Y. Liu, W. Shu, H. Luo, S. Wen, Generation of arbitrary vector vortex beams on hybrid-order poincaré sphere, Photon. Res. 5 (2017) 15–21. doi:10.1364/PRJ.5.000015.

[47] M. Erhard, R. Fickler, M. Krenn, A. Zeilinger, Twisted photons: new quantum perspectives in high dimensions, Light. Sci. Appl. 7 (17146) (2018). doi:10.1038/lsa.2017.146.

[48] P. A. M. Dirac, The quantum theory of the electron, Proc. R. Sco. Lond. A 1117 (1928) 610–624. doi:10.1098/rspa.1928.0023.

[49] S. M. Barnett, R. P. Cameron, A. M. Yao, Duplex symmetry and its relation to the conservation of optical helicity, Phys. Rev. A 86 (2012) 013845. doi:10.1103/PhysRevA.86.013845.

[50] J. Bardeen, L. N. Cooper, J. R. Schrieffer, Theory of superconductivity, Phys. Rev. 108 (1957) 1175–1204. doi:10.1103/PhysRev.108.1175.

[51] J. R. Schrieffer, Theory of Superconductivity, CRC Press, Boca Raton, 1971. doi:10.1201/9780429495700.

[52] S. Tomonaga, Remarks on Bloch's method of sound waves applied to many-fermion problems, Prog. Theor. Phys. 5 (1950) 544–569. doi:10.1143/PTP.5.544.

[53] J. M. Luttinger, An exactly soluble model of a many-fermion system, J. Math. Phys. 4 (1963) 1154–1162. doi:http://dx.doi.org/10.1063/1.1704046.

[54] T. Giamarchi, Quantum Physics in One Dimension, Oxford University Press, Oxford, 2004.

[55] P. W. Anderson, Random-phase approximation in the theory of superconductivity, Phys. Rev. 112 (1958) 1900–1916. doi:10.1103/PhysRev.112.1900.

[56] N. N. Bogoljubov, On a new method in the theory of superconductivity, IL Nuovo Cimento 7 (1958) 794–805. doi:10.1007/BF02745585.

[57] S. Pancharatnam, Generalized theory of interference, and its applications, Proc. Indian Acad. Sci., Sect. A XLIV (1956) 398–417. doi:10.1007/BF03046050.

[58] M. V. Berry, Quantual phase factors accompanying adiabatic changes, Proc. R. Sco. Lond. A 392 (1984) 45–57. doi:10.1098/rspa.1984.0023.

[59] A. Tomita, R. Y. Cao, Observation of Berry's topological phase by use of an optical fiber, Phys. Rev. Lett. 57 (1986) 937–940. doi:10.1103/PhysRevLett.57.937.

[60] R. Simon, N. Mukunda, Bargmann invariant and the geometry of güoy effect, Phys. Rev. Lett. 70 (1993). doi:10.1103/PhysRevLett.70.880.

[61] K. Bliokh, Geometrodynamics of polarized light: Berry phase and spin Hall effecct in a gradient-index medium, J. Opt. A: Pure Appl Opt. 11 (2009) 094009. doi:10.1088/1464-4258/11/9/094009.

[62] Y. Nambu, Quasi-particles and gauge invariance in the theory of superconductivity, Phys. Rev. 117 (1960) 648–663. doi:10.1103/PhysRev.117.648.

[63] J. Goldstone, A. Salam, S. Weinberg, Broken symmetries, Phy. Rev. 127 (1962) 965–970. doi:10.1103/PhysRev.127.965.

[64] P. W. Higgs, Broken symmetries, massless particles and gauge fields, Phys. Lett 12 (1962) 132–133. doi:10.1103/PhysRevLett.13.508.
